# Astrocytic stress response is induced by exposure to astrocyte-binding antibodies expressed by plasmablasts from pediatric patients with acute transverse myelitis

**DOI:** 10.1186/s12974-024-03127-2

**Published:** 2024-06-24

**Authors:** Chad Smith, Kiel M. Telesford, Sara G. M. Piccirillo, Yamhilette Licon-Munoz, Wei Zhang, Key M. Tse, Jacqueline R. Rivas, Chaitanya Joshi, Dilan S. Shah, Angela X. Wu, Ritu Trivedi, Scott Christley, Yu Qian, Lindsay G. Cowell, Richard H. Scheuermann, Ann M. Stowe, Linda Nguyen, Benjamin M. Greenberg, Nancy L. Monson

**Affiliations:** 1 UT Southwestern Department of Neurology, Dallas, TX USA; 2 The Brain Tumor Translational Laboratory, Department of Cell Biology and Physiology, University of New Mexico Health Sciences Center, Albuquerque, NM USA; 3 University of New Mexico Comprehensive Cancer Center, Albuquerque, NM USA; 4 UT Southwestern O’Donnell School of Public Health, Dallas, TX USA; 5 J. Craig Venter Institute, La Jolla, CA USA; 6 National Library of Medicine, National Institutes of Health, Bethesda, MD USA; 7 Department of Neurology, University of Kentucky, Lexington, KY USA; 8 UT Southwestern Department of Immunology, Dallas, TX USA

## Abstract

**Background:**

Pediatric acute transverse myelitis (ATM) accounts for 20–30% of children presenting with a first acquired demyelinating syndrome (ADS) and may be the first clinical presentation of a relapsing ADS such as multiple sclerosis (MS). B cells have been strongly implicated in the pathogenesis of adult MS. However, little is known about B cells in pediatric MS, and even less so in pediatric ATM. Our lab previously showed that plasmablasts (PB), the earliest B cell subtype producing antibody, are expanded in adult ATM, and that these PBs produce self-reactive antibodies that target neurons. The goal of this study was to examine PB frequency and phenotype, immunoglobulin selection, and B cell receptor reactivity in pediatric patients presenting with ATM to gain insight to B cell involvement in disease.

**Methods:**

We compared the PB frequency and phenotype of 5 pediatric ATM patients and 10 pediatric healthy controls (HC) and compared them to previously reported adult ATM patients using cytometric data. We purified bulk IgG from the plasma samples and cloned 20 recombinant human antibodies (rhAbs) from individual PBs isolated from the blood. Plasma-derived IgG and rhAb autoreactivity was measured by mean fluorescence intensity (MFI) in neurons and astrocytes of murine brain or spinal cord and primary human astrocytes. We determined the potential impact of these rhAbs on astrocyte health by measuring stress and apoptotic response.

**Results:**

We found that pediatric ATM patients had a reduced frequency of peripheral blood PB. Serum IgG autoreactivity to neurons in EAE spinal cord was similar in the pediatric ATM patients and HC. However, serum IgG autoreactivity to astrocytes in EAE spinal cord was reduced in pediatric ATM patients compared to pediatric HC. Astrocyte-binding strength of rhAbs cloned from PBs was dependent on somatic hypermutation accumulation in the pediatric ATM cohort, but not HC. A similar observation in predilection for astrocyte binding over neuron binding of individual antibodies cloned from PBs was made in EAE brain tissue. Finally, exposure of human primary astrocytes to these astrocyte-binding antibodies increased astrocytic stress but did not lead to apoptosis.

**Conclusions:**

Discordance in humoral immune responses to astrocytes may distinguish pediatric ATM from HC.

**Supplementary Information:**

The online version contains supplementary material available at 10.1186/s12974-024-03127-2.

## Background

Pediatric acute transverse myelitis (ATM) is an immune-mediated inflammatory disease of the spinal cord with an incidence rate of 1.7–2 pediatric cases per million children per year [7, 13, 46]. ATM typically presents with limb weakness, sensory deficits, and/or bladder/bowel dysfunction evolving over hours to days. Magnetic resonance imaging (MRI) is the primary tool for diagnosis of ATM, particularly as cerebrospinal fluid (CSF) protein and cell counts can be normal in 20–50% of pediatric ATM cases [2]. ATM accounts for 20–30% of children presenting with a first acquired demyelinating syndrome, and can occur as an isolated syndrome, known as idiopathic TM. However, it can also be the first clinical presentation of other acquired demyelinating syndromes (ADS), including multiple sclerosis (MS), aquaporin-4-IgG positive neuromyelitis optica spectrum disorder (NMOSD) or myelin oligodendrocyte glycoprotein antibody-associated disease (MOGAD) diagnosis [3, 7].

Compared to adults, children with ATM appear to have a lower incidence of MS. Epidemiological studies have shown approximately one-third of adult patients presenting with ATM evolve to a diagnosis of MS [15, 22, 28, 35, 39] while only 22% of pediatric patients evolve to a diagnosis of MS [8, 18]**.** Additionally, compared to adults, children usually improve within 2 weeks following acute immunotherapy (eg corticosteroids, IVIG and plasmaexchange) and have better outcomes, with nearly one-half making a complete recovery by 2 years [2]. One possible difference could be variations in the composition of lymphocytes involved in the autoimmune pathology of children compared to adults.

B cells in particular, have been implicated in the pathogenesis of CNS autoimmune diseases, including MS. CSF or blood B cell biomarkers to identify the extent of neuronal and glia inflammation, stress and irreversible injury are however lacking in ATM. We previously demonstrated that plasmablasts (PBs), the earliest B cell subtype producing antibody, are expanded in adults with ATM [30]. Plasmablasts are a recently activated antigen-experienced B cell subtype whose default program is to produce high affinity neutralizing antibodies against non-self antigens [4], mature into long-lived plasma cells, and home to the bone marrow where they can produce antibodies for decades [38]. Autoreactive PBs are typically undetectable in healthy individuals, but in the context of adult ATM, 28% of PBs that we isolated from the expanded PB pool produce self-reactive antibodies that target cytoplasmic antigens within neurons. These autoreactive PBs also tend to utilize antibody variable heavy chain 4 (VH4) family genes.

Little is known about B cell involvement in pediatric ATM, although altered B cell subset distributions are reported in pediatric MS [44]. Therefore, we investigated pediatric ATM patients and healthy controls to profile B cells for evidence of involvement in CNS autoimmunity associated with pediatric ATM. We found that the frequency of peripheral blood PBs was reduced in pediatric ATM patients compared to pediatric HC. Pediatric ATM patients had a similar frequency of VH4 + PB compared to pediatric HC. Most autoreactive PBs from pediatric ATM patients recognized astrocytes in CNS tissue and primary human astrocytes in cell culture. Accumulation of somatic hypermutation into the antibody heavy chain genes increased binding intensity to astrocytes. Exposure of human primary astrocytes to astrocyte-binding antibodies cloned from PBs of pediatric ATM subjects induced astrocytic stress, but did not advance to apoptotic events.

## Materials and methods

### Human blood acquisition and processing

All subjects and/or their legally authorized study partners signed the written informed consent approved by the Institutional Review Board of the UT Southwestern Medical Center (UTSW), in accordance with the Federal-wide Assurance on file with the Department of Health and Human Services (USA). The age, gender, diagnosis, and other patient information is reported in Table 1. Treatment-naïve pediatric ATM patients at high risk for developing MS (ages 3–13) and pediatric healthy controls (HC) (ages 3–12) were included and were matched by age. As pediatric ATM cases are rare, no power analysis was performed to determine the number of subjects needed to detect a difference between the groups. Pediatric patients were classified as high risk for MS if they presented with at least one non-enhancing white matter lesion by MRI and/or a high Ig index in the cerebrospinal fluid. None of these patients had evidence of autoantibody titers to MOG or AQP4. We collected whole blood in Acid Citric Dextrose (ACD) tubes for this study. After collection, ACD tubes were centrifuged to collect plasma and the non-plasma portion subjected to Ficoll centrifugation to isolate peripheral blood mononuclear cells (PBMCs). Plasma was stored in aliquots at -80˚C and PBMCs were stored in aliquots at -140˚C. Immunogenetics of pediatric TM and HC were compared to adult CIS patients at high risk for developing MS presenting with transverse myelitis, which were reported previously [39].

**Table 1:** Pediatric subject information

Patient code	Age	Gender	Current Diagnosis	Oligoclonal band status	Ig index
PTM-A	12	F	ATM	0	Unknown
PTM-B	4	F	ATM	0	0.63
PTM-C	13	F	ATM	0	0.59
PTM-D	10	F	ATM	0	0.47
PTM-E	13	F	ATM	Unknown	Unknown
PHC-A	12	F	Healthy	NA	NA
PHC-B	6	F	Healthy	NA	NA
PHC-C	9	F	Healthy	NA	NA
PHC-D	5	F	Healthy	NA	NA
PHC-E	4	F	Healthy	NA	NA
PHC-F	11	M	Healthy	NA	NA
PHC-G	10	M	Healthy	NA	NA
PHC-H	10	M	Healthy	NA	NA
PHC-I	7	F	Healthy	NA	NA
PHC-J	3	F	Healthy	NA	NA

### Mass cytometry (CyTOF)

Cryopreseved PBMCs were thawed in complete RPMI, counted, filtered, and treated with cisplatin (2.5μM, Enzo Life Sciences, Farmingdale, NY, USA) in serum free RPMI to confirm viability [14]. Cell staining for mass cytometry was performed as described previously [9], using antibody panels in table S6. Samples were mass-channel barcoded, pooled, and stained as a single sample [56]. Readouts were recorded on a CyTOF2 mass cytometer (Fluidigm, South San Francisco, CA, USA). Data were analyzed in Cytobank (cytobank.org) and with FlowJo software (flowjo.com), including viSNE [6], and FlowSOM [49] analysis.

### Single-cell antibody immunogenetics

Individually sorted antigen-experienced CD19 + CD27 + CD38+ B cells from the blood were lysed, mRNA was reverse-transcribed, and immunoglobulin variable regions were amplified with multiple rounds of PCR as previously described (32). Sanger sequencing was performed at the UTSWMC (University of Texas Southwestern Medical Center) sequencing core to generate the antibody variable domain reads. Sequences were analyzed using the VDJServer analysis portal (33). V, D, J gene calls, FR and CDR regions were annotated using IgBlast v1.14.1 (34) with VDJServer IMGT 2019.01.23 germline database.

### Recombinant human antibody cloning, expression, and isolation

HEK293T cells (ATCC, Manassas, VA) were maintained in HyClone Dulbeccos Modified Eagles Medium (DMEM) (GE Healthcare Life Sciences). All recombinant human antibodies (rhAbs) were transiently transfected into HEK293T cells with the lipid transfection reagent JetPEI (PolyPlus Transfection) as done previously [29, 39]. Supernatants from these cultures were collected on days 3, 5, 7 and 10. The cell pellets were spun down and supernatants were passed through 0.2um filters and subjected to antibody purification on the NGC QUEST 10 system. The concentration of the antibodies were determined by sandwich ELISA as done previously [29, 37–39].

### Purification of IgG from plasma

To purify bulk IgG from plasma samples obtained from ACD whole blood collections, we employed protein G chromatography (Cytiva Life Sciences). We quantified pure IgG preparations with an in-house IgG ELISA to facilitate normalization across all samples for assays.

### Fluorescent immunohistochemistry

At the height of EAE symptoms, mice were perfused transcardially with PBS and 4% PFA, and their brains and spinal cords were extracted. After a further 48 h post-fix with 4% PFA, tissue was washed with PBS and embedded in paraffin. Sagittal brain slices and transverse cord slices (5 μm) were obtained by the UT Southwestern Histo Pathology Core. Slices were deparaffinated with 2 × 10 min washes with xylenes (Fisher), then washed with successive ethanol solutions: 2 × 100% ethanol, 95% ethanol, 70% ethanol, 50% ethanol, 30% ethanol. Slices were washed with PBS for 3 min, then 1% Glycine (Sigma) for 15 min. Slices underwent antigen retrieval by submersion in boiling Citrate Retrieval Buffer (Vector BioLabs H-3300) in a pressure cooker for 5 min. Slides were washed 2 × 5 min with PBS, then with 0.2% Triton X-100 (Sigma) for 10 min. Slides were blocked for 2 h with 5% Goat Serum (ThermoFisher 50062Z) + 0.1% Triton X-100 in a humidified chamber, then incubated overnight at 4C in a humidified chamber with primary antibodies diluted in blocking buffer: rhAbs (20 μg/mL), purified human IgG (2 μg/mL), Rabbit anti-GFAP (1:100 Abcam ab16997), Mouse anti-MAP2 (1:200 ThermoFisher MA5-12826). The next day, slides were washed 3 × 5 min with 0.025% Triton X-100, then incubated for 1 h at RT in a humidified chamber with secondary antibodies diluted in blocking buffer: Goat anti-Human conj AlexaFluor 488 (1:1000 ThermoFisher A-11013), Goat anti-Rabbit conj AlexaFluor 568 (1:1000 Abcam ab175471), Goat anti-Mouse conj AlexaFluor 647 (1:500 Abcam ab150115). The rinses were repeated, then slides were counterstained with DAPI. Slides were washed twice with PBS and coverslips were mounted with Fluoromount G (ThermoFisher 00-4958-02). Slides were visualized on a Zeiss LSM780 confocal microscope with a 40 ×/1.45NA oil immersion lens.

### Human astrocytes

Human astrocytes were purchased from ScienCell Research Laboratories (Carlsbad, CA), grown in DMEM (Sigma-Aldrich, St. Louis, MO, USA) and supplemented with 10% heat-inactivated fetal bovine serum (Gemini Bio-Products, Sacramento, CA, USA), 1% 100 × Penicillin–Streptomycin (Sigma-Aldrich, St. Louis, MO, USA) and 1% 200 mM l-glutamine (Sigma-Aldrich, St. Louis, MO, USA).

### Fluorescent immunocytochemistry

Cells were plated on laminin-coated coverslips (Sigma L2020) and rested overnight in an 37 °C/5% CO_2_ incubator. The next day, cells were fixed with ice-cold 4% PFA for 10 min, then washed with PBS for 5 min. Cells were washed with 0.2% Triton X-100 + 2 mg/mL BSA (Sigma A9647) for 10 min, then blocked with 0.1% BSA + 1% Goat Serum + 3% BSA for 2 h. Cells were incubated overnight at 4 °C with primary antibodies diluted in blocking buffer: rhAbs (20 μg/mL), Rabbit anti-GFAP (1:200). The next day, cells were washed 4 × 3 min with 0.05% Triton X-100 + 1% Goat Serum + 1% BSA, then incubated for 1 h at RT with secondary antibodies diluted in blocking buffer: Goat anti-Human conj AlexaFluor 488, Goat anti-Rabbit conj AlexaFluor 568. The washes were repeated, then cells were counterstained with DAPI and washed twice with PBS. Coverslips were mounted on glass slides with Fluoromount G, then visualized on a Zeiss Axioscope.A1 fluorescent microscope with a 20×/0.45NA lens.

### Commercial cell based assays

For measuring reactivity of rhAbs to AQP-4, we used cell-based assays according to the manufacturer’s instructions (Euroimmun FA1128-1010-50). This assay expresses AQP-4 in fixed HEK293 cells [52]. For measuring reactivity to Hep-2 cells, we used a cell-based assay according to the manufacturer’s instructions (Aesku.Bion ANK-120). This assay consists of fixed Hep-2 cells. In both assays, the rhAbs were diluted to 20 μg/mL in 0.1% Tween-20 (Fisher) + 1% Goat Serum and cells were counterstained with DAPI. Cells were imaged as described above and quantified with FIJI. Positive staining of a rhAb was defined as fluorescence intensity equal to or greater than the mean fluorescent intensity of the negative control plus 20 standard deviations.

### Internalization procedure

After plating cells and resting overnight, rhAbs were diluted in 6 mM Glucose (Sigma) + 1% BSA + 2% Fetal Bovine Serum + 1 mM Pyruvate (Gibco 111360-070) and added to cell cultures at 20 μg/mL. After incubation, cells were washed 3 × with ice-cold PBS, then fixed with 2% PFA + 0.5% Triton X-100 for 2 min. Of note, exposure of the cells to Triton X-100 does not occur until after the internalization step is complete. Cells were fixed for an additional 15 min with 4% PBS, then washed 3 × with PBS. Cells were blocked with 0.01% saponin (Sigma 47036) + 2% BSA + 0.1% lysine (Sigma 62840) for 30 min, then incubated with primary antibodies in blocking buffer for 1 h: Rabbit anti-Clathrin (1:100 Cell Signaling 4796S), Mouse anti-EEA1 (1:100 Cell Signaling 48453S). Cells were washed 6 × 5 min with blocking buffer, then incubated with secondary antibodies and DAPI in blocking buffer for 1 h: Goat anti-Human conj AlexaFluor 488, Goat anti-Rabbit conj AlexaFluor 568, Goat anti-Mouse conj AlexaFluor 647. Cells were washed 6 × 5 min with PBS, then fixed for 10 min with 4% PFA and washed 2 more times. Coverslips were mounted with FluoroMount G and visualized with a Nikon CSU-W1 spinning disk confocal microscope.

### Quantification of autoreactivity by rhAbs

Autoreactivity was quantified using Fiji [43]. Cells were identified by using the Threshold function to detect nuclei and were added to the Region of Interest (ROI) manager. The mean fluorescence intensity (MFI) of positively-stained cells was measured, and the MFI of the background (regions where no cells were detected) was subtracted from this value.

### Toxicity procedure

rhAbs were added to cell cultures as described above. Human astrocytes were incubated for 6 h prior to heat shock or remaining at 37 °C for 1.5 h. Cells were incubated with 6 μM Caspase-3/7 Green Detection Reagent (Invitrogen C10423) for the last 30 min of the incubation. Cells were fixed with 4% PFA then underwent immunocytochemistry with the following antibodies: Mouse anti-G3BP1 (1:100 BD Biosciences 611126), Goat anti-Human conj AlexaFluor 568 (1:1000 ThermoFisher A-21090), Goat anti-Mouse conj AlexaFluor 647. Coverslips were mounted and visualized with a Nikon CSU-W1 spinning disk confocal microscope.

### Statistical analyses

Analyses were performed with GraphPad Prism 7 software (GraphPad Software, Inc., San Diego, CA, USA). One-way ANOVA was used to compare flow cytometry and CyTOF data, unless there were less than three groups in which case a Student’s T-test was appropriate. For immune repertoire data additional corrections were performed for multiple comparisons of frequencies [39]. Frequencies subjected to arcsine transformation and non-parametric ANOVA was used with a post-hoc analysis (the Dunnett multiple comparison method) for pairwise comparison of patient groups with healthy donors.

## Results

### Patient plasmablast frequency correlates with age

**Fig. 1:**
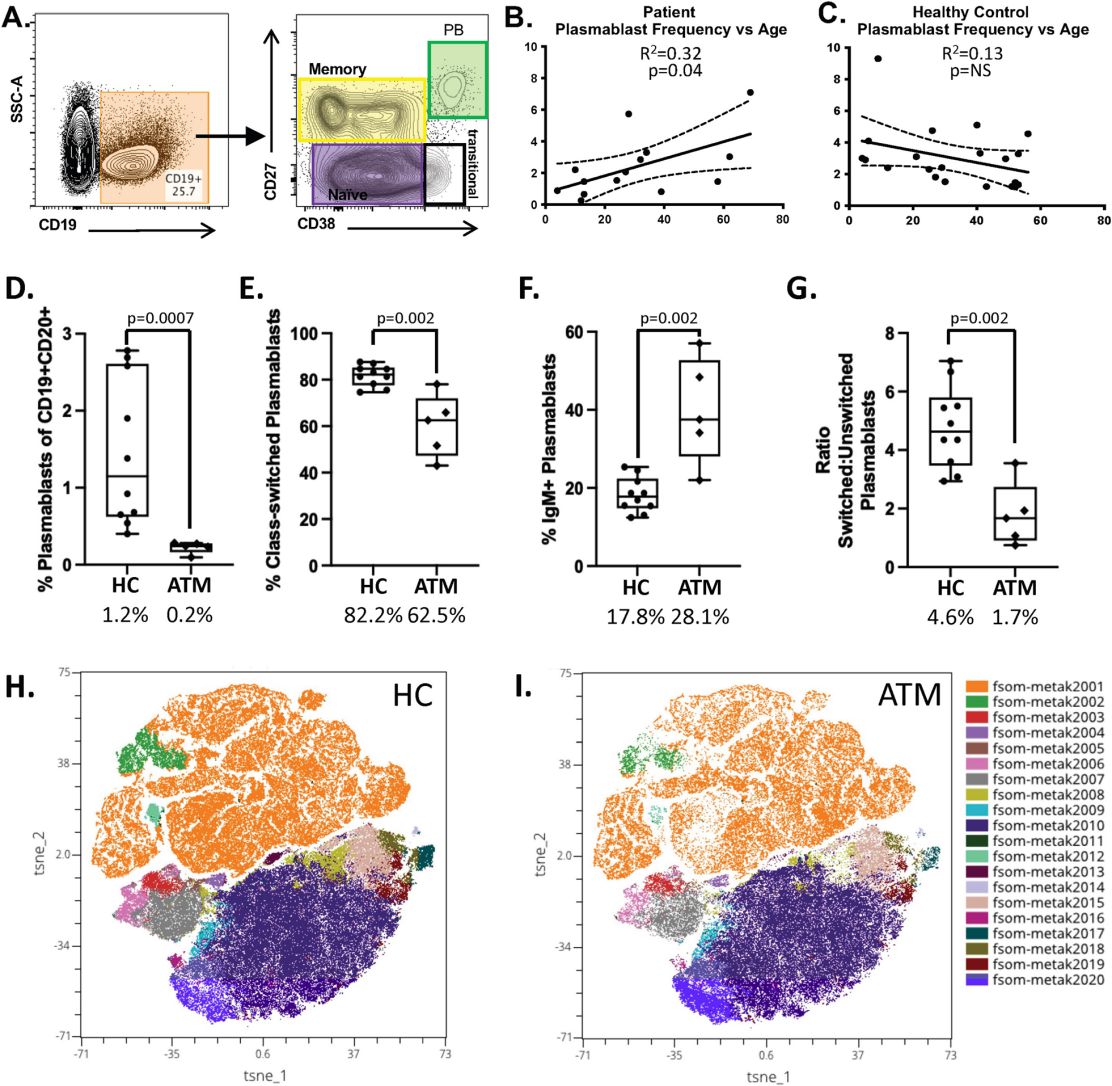
Profiling of B cell subsets. **A** Gating strategy to obtain the frequency of plasmablast (PB) in each sample using traditional flow cytometry. **B** Plasmablast frequency correlation with age in patients with acute transverse myelitis using traditional flow cytometry. **C** Plasmablast frequency correlation with age in healthy controls using traditional flow cytometry. **D–G** Plasmablast subset frequencies in pediatric acute transverse myelitis and healthy controls using CyTOF data. **H, I** TSNE plots of CyTOF data identifying 20 distinct B cell subsets in the cohort

Adult patients presenting with ATM symptoms demonstrate an increased frequency of CD19^+/low^CD27^HI^ plasmablasts in the blood compared to controls [30]. We hypothesized that pediatric patients presenting with ATM symptoms would also demonstrate an increased frequency of plasmablasts in the blood. To investigate this, we used conventional flow cytometry to identify (Fig. 1A) and calculate the frequency of plasmablasts in the blood of 5 pediatric ATM patients and 10 pediatric healthy controls (HC). While the frequency of plasmablasts in the blood of the pediatric ATM patients and pediatric HC was no different (2.1% vs 2.5%, p = NS) (data not shown), we did note that the frequency of plasmablasts in the blood of pediatric and adult ATM patients increases with age (Fig. 1B) whereas plasmablasts in the blood of pediatric and adult HC does not (Fig. 1C).

### Pediatric patient plasmablast frequency is reduced compared to HC

To further our understanding of this early immune profiling data, we subjected the pediatric cohort (5 TM patients and 10 HC) to CyTOF technology which provided frequency information on 15 B cell subsets using a traditional gating strategy (Supplemental Fig. 1A and Supplemental Table 1). This data demonstrates that the overall frequency of plasmablasts in pediatric HC is significantly higher compared to pediatric ATM patients (p = 0.0007; Fig. 1D). Class-switched plasmablasts constituted the majority of the plasmablast population in pediatric HC (p = 0.002; Fig. 1E), while the frequency of IgM + plasmablasts was higher in pediatric ATM (Fig. 1F), resulting in a switched:unswitched plasmablast ratio that was higher in the pediatric HC compared to ATM (Fig. 1G). Next, we used FlowSOM analysis on the OMIQ platform comparing pediatric ATM and HC cohorts using the CD3^−^CD14^−^ live singlets (Supplemental Fig. 1B) as the input to the FlowSOM clustering which generated 20 unique B cell clusters in the dataset (Fig. 1H, I). Of those, Cluster 12 (CD27^+^CD38^+^ plasmablasts) displayed discordant frequency between the ATM and HC cohorts, confirming our findings by traditional gating of the dataset. Cluster 13 (IgM^+^CD22^+^) was also noted to display discordant frequency between the ATM and HC cohorts.

### Pediatric patient serum IgG displays autoreactivity

**Fig. 2:**
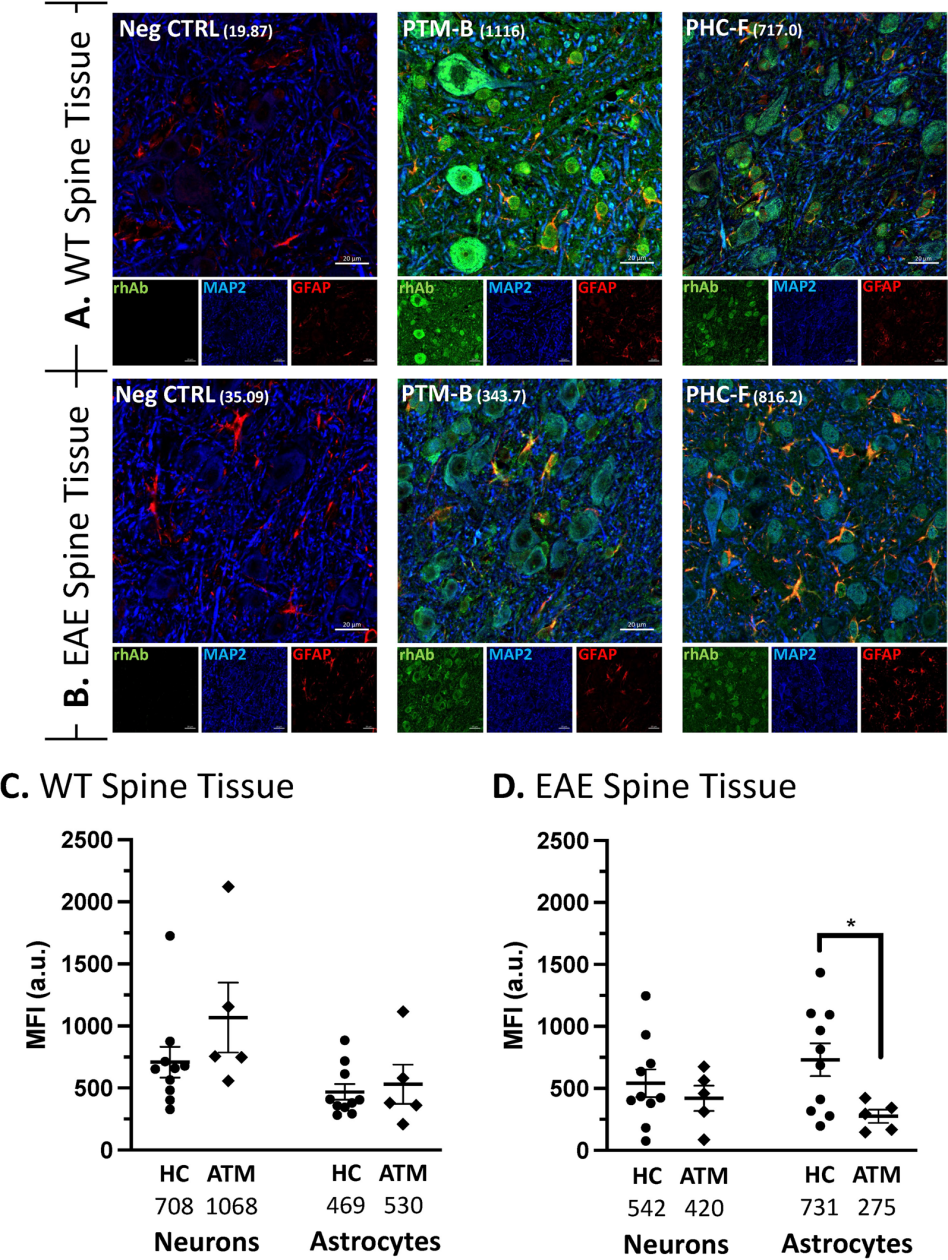
IgG purified from serum samples bind neurons and astrocytes in mouse spinal cord. **A, B** Representative images from the lumbar spinal cord of WT (**A**) and EAE (**B**) mice. Green: IgG staining. Red: GFAP. Blue: MAP2. In the merge panels, yellow indicates co-stain of GFAP with the rhAb. The MFI of astrocytic staining is indicated in parentheses. Scale bar: 20 μm. **C, D** Scatter plots of the mean fluorescent intensity of neuron and astrocyte binding in WT (**C**) and EAE (**D**) tissue using IgG purified from patient and control serum. The average MFI of each cohort is provided below the x-axis

Plasmablasts, and more specifically, class-switched plasmablasts were not expanded in the pediatric ATM patients, but the antibodies produced by this reduced fraction of plasmablasts may be enriched for autoreactivity. To address this, we asked whether serum IgG from these patients displayed binding to neurons or glia in spinal cord tissue. We tested for binding on mouse spinal cord since transverse myelitis symptoms originate with pathology in this tissue. We used spinal cord from wild type (WT) mice with no evidence of neuroinflammation (Fig. 2A and Supplementary Fig. 2) and spinal cord from mice that had experienced hind limb paralysis due to neuroinflammation following induction of Experimental Autoimmune Encephalitis (EAE) (Fig. 2B and Supplementary Fig. 2). Using wild type spinal cord, we did not observe discordance in mean MFI binding to neurons or astrocytes between serum IgG from the pediatric ATM and HC groups (Fig. 2C). Serum IgG samples from these groups also demonstrated similar mean MFI binding in neurons of EAE spinal cord (Fig. 2D). However, the mean MFI binding of astrocytes by pediatric ATM serum IgG was significantly reduced in EAE spinal cord compared to mean MFI astrocyte binding by pediatric HC serum IgG (Mean MFI 731 vs 275, p = 0.039) (Fig. 2D).

### Pediatric patient plasmablast discordance in heavy chain family use

The observations of reduced plasmablast frequency and reduced mean MFI binding to astrocytes in EAE spinal cord by serum IgG of these pediatric ATM patients compared to pediatric HC prompted us to examine potential discordance in antibody gene distribution between pediatric ATM and HC. To do this, we sorted plasmablasts from the blood of pediatric ATM patients and HC, then used our single cell PCR pipeline to obtain the frequency of heavy chain family usage among the plasmablast populations of each subject. Adult ATM patients display a robust expansion of VH4 family use compared to controls [30], but we did not observe enrichment of VH4 family use in the pediatric ATM patients compared to pediatric HC (Table 2). VH3 family use in the pediatric ATM patients was significantly reduced in comparison to pediatric HC (p = 0.016), but this did not alter the VH4:VH3 ratio of pediatric ATM patients compared to HC. The plasmablasts from pediatric ATM patients did display a higher mutation accumulation in comparison to pediatric HC (p = 0.047), but this was not specific to plasmablasts from pediatric ATM patients expressing VH4 family genes.

**Table 2:** Antibody heavy chain profile

	HC	ATM	P-value
Percent VH4	26.02 ± 11.62	30.76 ± 12.74	NS
Percent VH3	73.24 ± 11.79	49.87 ± 9.9	P = 0.016
VH4:VH3	0.39 ± 0.24	0.68 ± 0.36	NS
Percent highly mutated plasmablasts	9%	23%	P = 0.047
Percent highly mutated VH4 + plasmablasts	17%	22%	NS

### Low prevalence of neuron binding to EAE spinal cord by VH3 + and VH4 + antibodies from pediatric subjects

To further our understanding of whether plasmablast immunogenetics influence binding properties of the antibodies they produce, we cloned antibodies expressed by 20 plasmablasts from the cohort in 4 categories: VH3 + with no mutations (VH3 + SHM−), VH3 + with high mutation accumulation (VH3 + SHM +), VH4 + with no mutations (VH4 + SHM−) and VH4 + with high mutation accumulation (VH4 + SHM +) (Supplemental Table 2). We cloned and expressed these antibodies as recombinant human antibodies (rhAbs) as previously described [29, 39] to determine reactivity. In each category, 2 rhAbs originated with pediatric HC and 3 rhAbs originated with pediatric ATM patients for a total of 20 rhAbs. We tested for binding on mouse spinal cord since transverse myelitis symptoms originate with pathology in this tissue. We used spinal cord from wild type (WT) mice with no evidence of neuroinflammation and spinal cord from mice that had experienced hind limb paralysis due to neuroinflammation associated with induction of Experimental Autoimmune Encephalitis (EAE). Of the 20 rhAbs tested, one bound neurons in WT spinal cord, 4 bound neurons in EAE spinal cord and one bound neurons in both WT and EAE spinal cord (Supplemental Table 2). Thus, 6 of the 20 rhAbs (30%) bound neurons in WT or EAE spinal cord. Of note, the two rhAbs that bound neurons in WT spinal cord were VH3+, while three of the five rhAbs that bound neurons in EAE spinal cord used VH4 antibody heavy chain genes. Both pediatric ATM and HC subjects had PBs producing neuron-binding rhAbs.

### Somatic hypermutation enhances glia binding to EAE spinal cord by VH3 + and VH4+ antibodies from pediatric ATM subjects

**Fig. 3:**
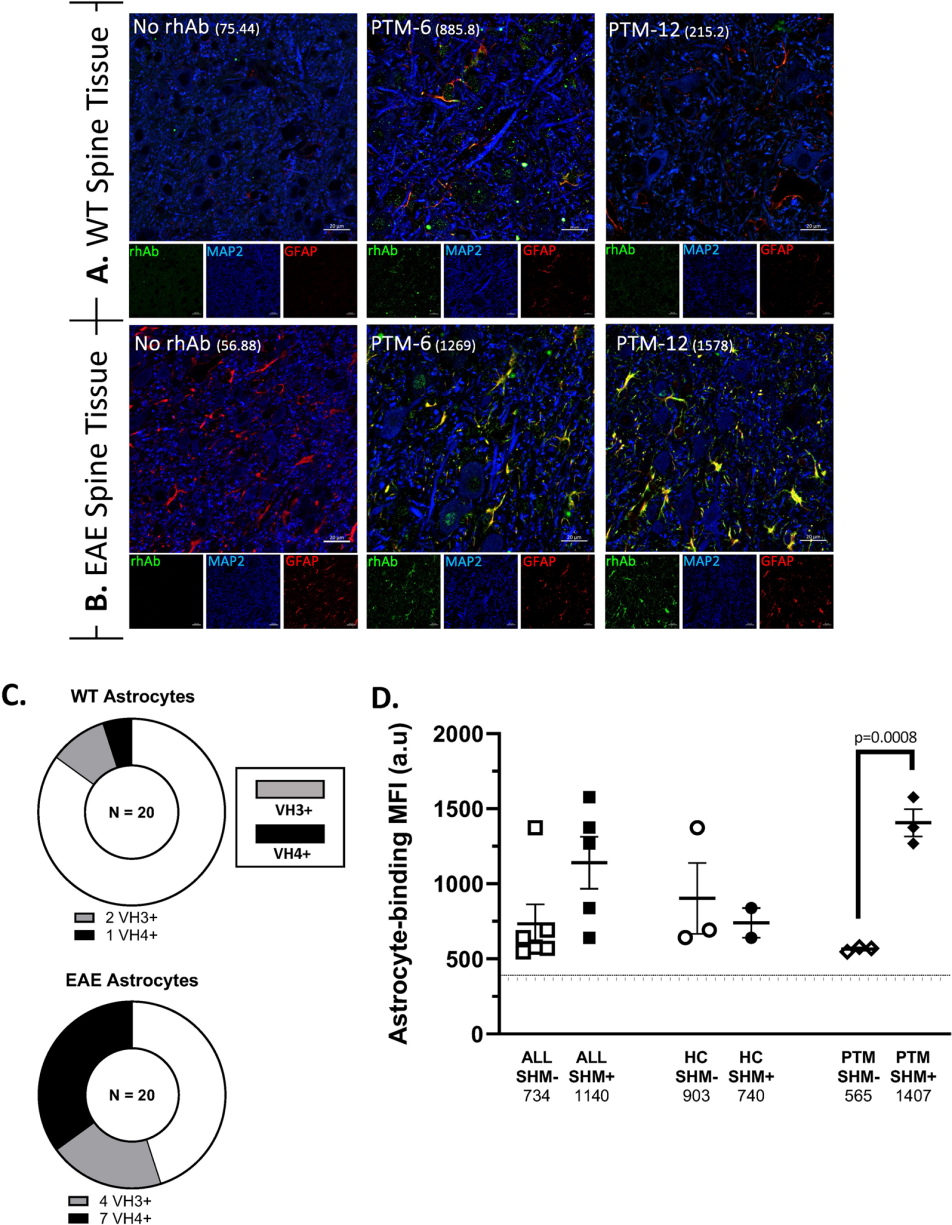
Plasmablast rhAbs bind neurons and astrocytes in mouse spinal cord. **A, B** Representative images of rhAbs binding neurons and astrocytes in the lumbar spinal cord of WT (**A**) and EAE (**B**) mice. Green: IgG staining. Red: GFAP. Blue: MAP2. In the merge panels, yellow indicates co-stain of GFAP with the rhAb. The MFI of astrocytic staining is indicated in parentheses. Scale bar: 20 μm. **C** Pie charts summarizing the number of rhAbs binding astrocytes in WT and EAE spinal cord tissue. **D** Scatter plots of the mean fluorescent intensity of the 11 astrocyte-binding rhAbs in EAE spinal cord tissue where the rhAbs are binned according to cohort (ALL, HC or ATM) and somatic hypermutation (SHM) accumulation status of the antibody heavy chain where “SHM−” indicates there is no SHM in the rhAb heavy chain and “SHM + ” indicates there is SHM in the rhAb heavy chain. Horizontal dashed line indicates the threshold of positivity which is set at 366.3. The average MFI of each cohort is provided below the x-axis

In contrast to the low frequency of neuron-binding rhAbs, we observed that 11 of the 20 rhAbs (55%) bound astrocytes in WT or EAE spinal cord (Fig. 3, Supplemental Fig. 3 and 4). All 5 of the EAE spinal cord neuron-binding rhAbs also bound astrocytes in EAE spinal cord (Supplemental Table 2). An additional 6 rhAbs acquired astrocyte binding in EAE spinal cord, but did not bind neurons in EAE spinal cord. Figures 3A, B display PTM-6 rhAb as an example of binding astrocytes in both WT and EAE spinal cord and PTM-12 rhAb as an example of binding astrocytes in EAE spinal cord only. Of the 11 that bound astrocytes in EAE spinal cord, 4 used VH3 family genes and 7 used VH4 family genes (Fig. 3C). Astrocyte binding intensity in EAE spinal cord was dependent on somatic hypermutation accumulation in the pediatric ATM cohort (Fig. 3D), since antibodies with somatic hypermutations bound astrocytes in EAE spinal cord with significantly higher mean MFI (2.5-fold) than antibodies without SHM (565 v 1407, p = 0.0008). The pediatric HC cohort had similar mean MFI binding to astrocytes in EAE spinal cord, independent of SHM accumulation.

**Fig. 4:**
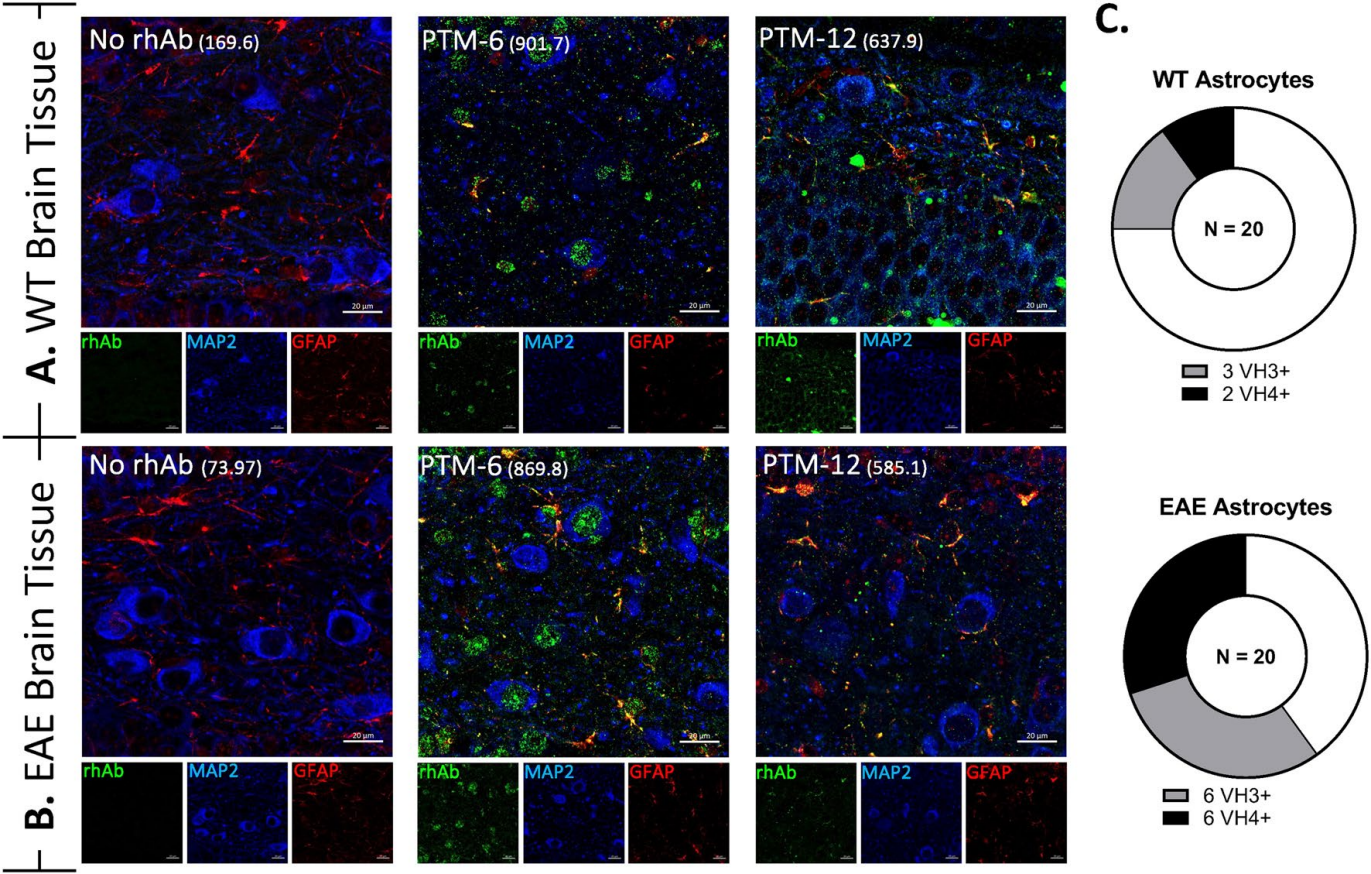
Plasmablast rhAbs bind neurons and astrocytes in mouse brain. **A, B** Representative images of rhAbs binding neurons and astrocytes in the brain of WT **(A)** and EAE (**B**) mice. Green: IgG staining. Red: GFAP. Violet: MAP2. Blue: DAPI. In the merge panels, yellow indicates co-stain of GFAP with the rhAb. The MFI of astrocytic staining is indicated in parentheses. Scale bar: 20 μm. **C** Pie charts summarizing the number of rhAbs binding astrocytes in WT and EAE spinal cord tissue. Positive binding rhAbs are binned according to antibody variable heavy chain use (VH3+ or VH4+)

To determine if binding was specific to astrocytes in spinal cord, we also investigated binding of the rhAbs to astrocytes in WT and EAE mouse brain (Fig. 4, Supplemental Table 2, and Supplemental Figs. 5 and 6). Of the 11 that bound astrocytes in EAE spinal cord, 9 bound astrocytes in EAE brain. Two of the 11 that bound astrocytes in EAE spinal cord did not bind astrocytes in EAE brain and one acquired astrocyte binding to EAE brain but did not bind astrocytes in EAE spinal cord. Figures 4A, B display the PTM-6 and PTM-12 rhAbs as examples of binding astrocytes in both WT and EAE brain. Thus, 5 of the 20 rhAbs (25%) displayed astrocyte binding in WT brain and 12 of the 20 rhAbs (60%) displayed astrocyte binding in EAE brain (Fig. 4C). Astrocyte binding in EAE brain by rhAbs was highly skewed towards those that originated from plasmablasts of pediatric HC (8 of 8 in HC compared to 4 of 12 in ATM). Astrocyte binding strength by rhAbs from pediatric HC in EAE brain was independent of SHM accumulation as we observed for EAE spinal cord (data not shown).

### Primary human astrocyte binding is similar in pediatric ATM patients and controls

**Fig. 5:**
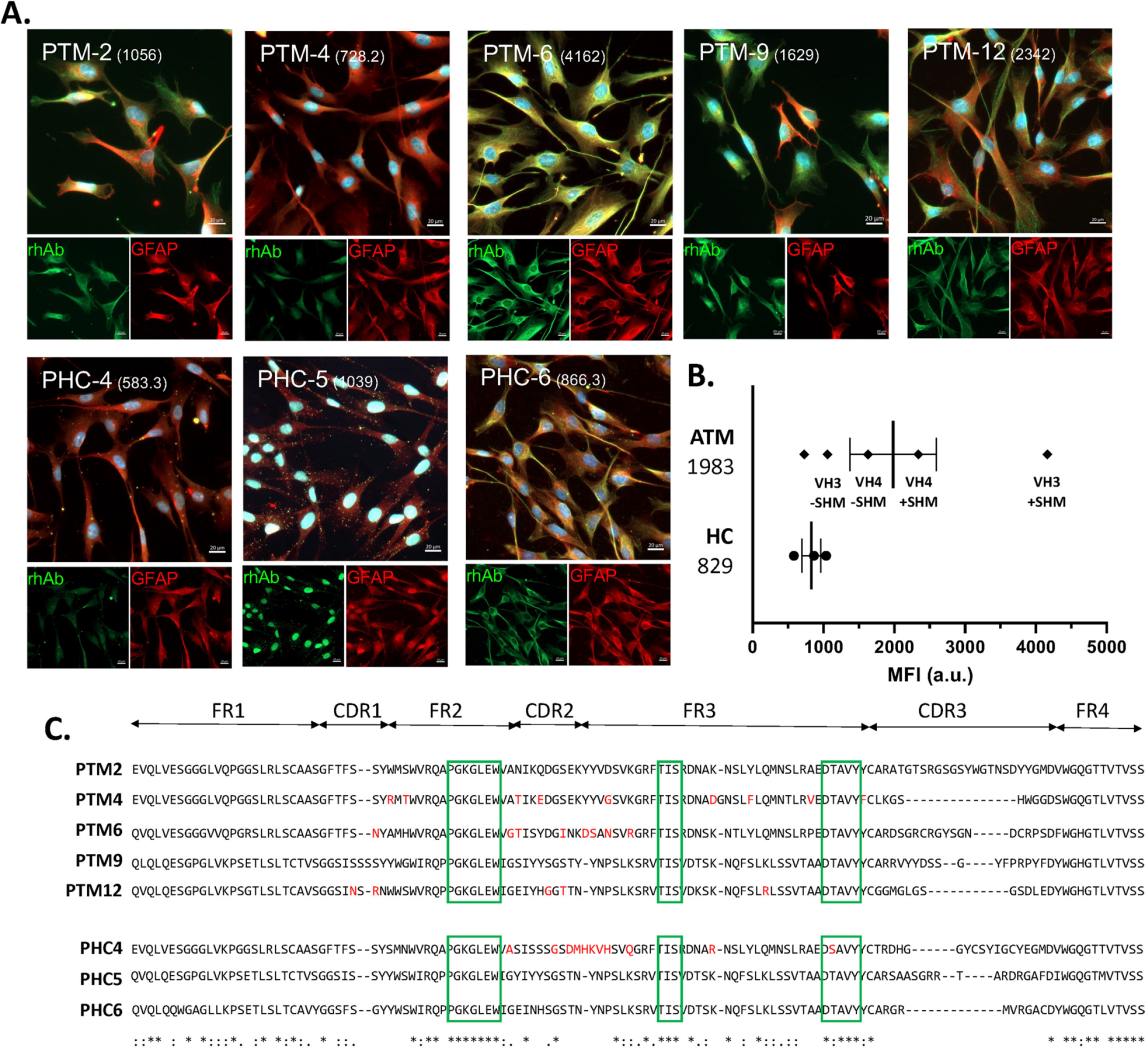
Plasmablast rhAbs bind to human primary astrocytes.** A** Representative images for the 8 rhAbs that bind primary human astrocytes. Green: rhAb staining. Red: GFAP. Blue: DAPI. In the merge panels, yellow indicates co-stain of GFAP with the rhAb. The MFI of astrocytic staining is indicated in parentheses. Scale bar: 20 μm. **B** Scatterplots of the MFI of human primary astrocytes where the rhAbs with positive binding are binned according to cohort (HC or ATM). The average MFI of each cohort is provided to the left of the x-axis. **C** Alignment of the antibody heavy chain sequences using Clustal Omega (EMBL-EBI). Only those sequences of rhAbs with positive binding to human primary astrocytes are shown and are binned according to cohort (ATM above and HC below). Green boxes demarcate the 3 motifs shared between astrocyte binding rhAbs. One letter codons that underwent replacement mutation are indicated in red letters. Codon positions which have a fully conserved residue in all sequences are indicated by “*”. Conservation between groups of strongly similar properties are indicated by “:”. Conservation between groups of weakly similar properties are indicated by “.”

Next, we investigated the binding properties of the 20 rhAbs to primary human astrocytes (Fig. 5, Supplemental Fig. 7). Of the 20 rhAbs tested, 8 bound to human primary astrocytes (Fig. 5A). Five of the 8 rhAbs that bound primary human astrocytes were from the pediatric ATM patients and the remaining 3 were from pediatric HC. Primary human astrocyte binding was observed with both VH3 + rhAbs and VH4 + rhAbs independent of SHM accumulation. The mean MFI of human primary astrocyte binding by rhAbs from the pediatric ATM cohort was similar to the mean MFI of human primary astrocyte binding by rhAbs from the pediatric HC cohort (Fig. 5B). No general immunogenetic features (including VH gene use, JH gene use, CDR3 length or charge) of the 5 rhAbs from pediatric ATM patients that bound primary human astrocytes displayed commonalities that might infer preference for astrocyte binding (Table 3). However, we did observe 3 motifs in FR2 and FR3 shared among rhAbs from either pediatric ATM or HC that bound human primary astrocytes (Fig. 5C). We also performed immunocytochemistry on the human epithelial cell line Hep-2 (Supplemental Fig. 8), a test commonly employed to detect anti-nuclear antibodies in SLE and a cell line expressing AQP4 (Supplemental Fig. 9), which identifies subjects that may be diagnosed with Neuromyelitis Optica. None of the rhabs in this cohort was reactive to AQP4 + HEK293 cells or Hep-2 cells.

**Table 3:** Gene information for primary human astrocyte positive rhAbs

rhAb name	rhAb category	VH gene JH gene	CDR3 AA length	CDR3 charge	VH #RM	VL gene JL gene	VL #RM	Patient
PTM-2	VH3 + SHM−	VH3-7 JH6	25	0	0	VK1-39 JK5	0	PTM-A
PTM-4	VH3 + SHM+	VH3-7 JH4	10	0	11	VK2-30 JK4	2	PTM-A
PTM-6	VH3 + SHM+	VH3-30 JH4	20	1	9	VK1-27 JK5	4	PTM-B
PTM-9	VH4 + SHM−	VH4-39 JH4	19	1	0	VK3-20 JK2	0	PTM-B
PTM-12	VH4 + SHM+	VH4-4 JH4	14	− 3	5	VK3-11 JK2	9	PTM-C

*VH* variable heavy, *JH* junction heavy, *CDR3* complementarity determining region 3, *AA* amino acid, *#RM* number of replacement mutations, *VL* variable light

### Astrocyte binding rhAbs from pediatric ATM patients enhance stress granule formation, but are not toxic to astrocytes

**Fig. 6:**
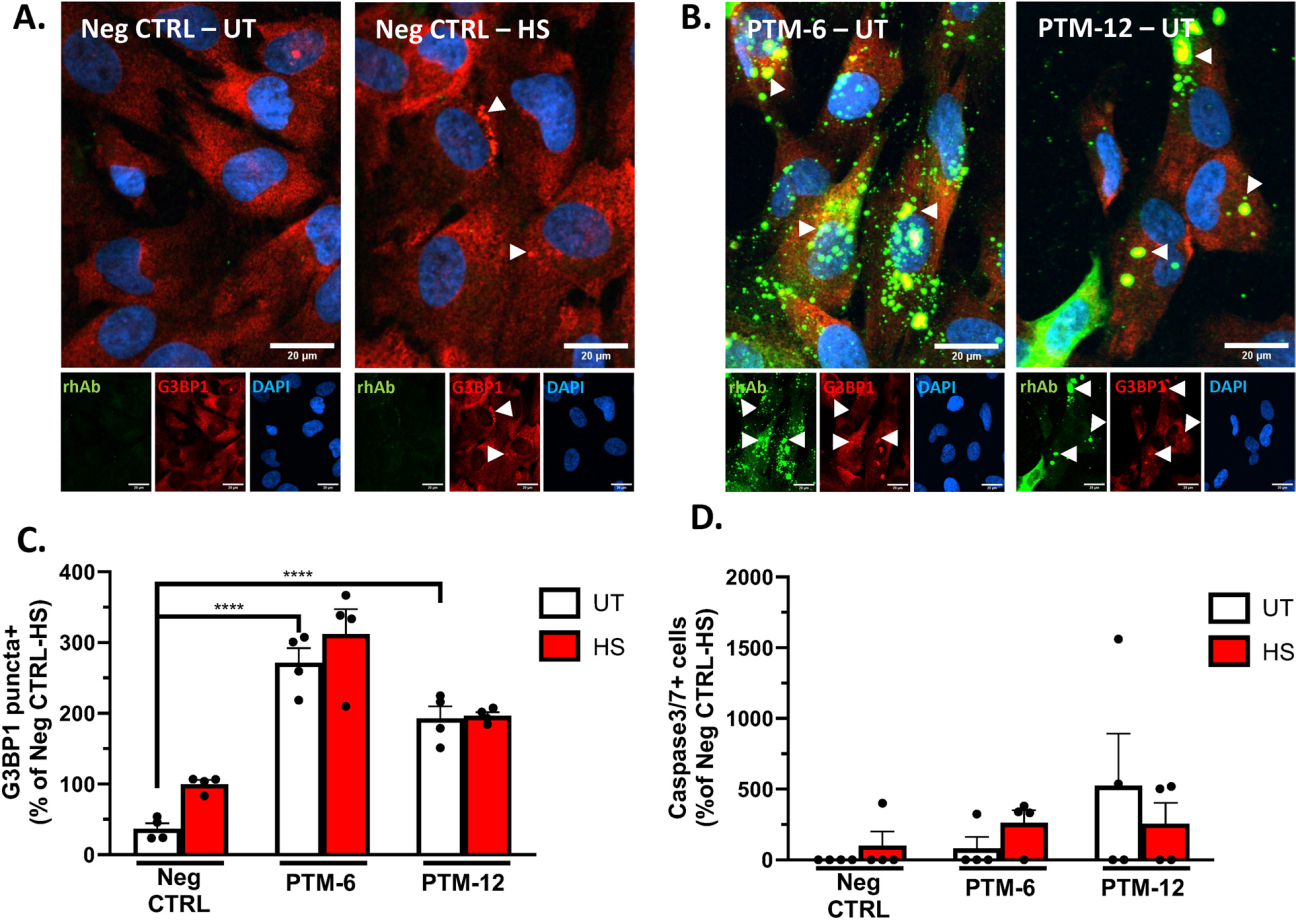
Astrocyte binding rhAbs induce astrocyte stress, but not apoptosis. **A** Representative images of untreated and heatshock treated human primary astrocytes to demonstrate G3BP1 + puncta formation. White arrowheads indicate G3BP1 + puncta. **B** Representative images of primary human astrocytes incubated with PTM-6 or PTM-12 rhAbs showing induction of G3BP1 + puncta. Green: rhAb. Red: G3BP1. Blue: DAPI. Violet: Active caspase 3/7. In the merge panels, yellow indicates co-stain of G3BP1 with the rhAb. Scale bar: 20 μm. **C** Summary statistics for G3BP1 puncta + cells as an indication of stress. **D** Summary statistics for Caspase 3/7 + cells as an indication of apoptosis

To investigate whether astrocyte binding impacts astrocyte health, we first determined the optimal time of rhAb internalization (Supplemental Fig. 10) in primary human astrocytes. Then the cultured primary human astrocytes were incubated with two of the rhAbs that displayed the highest intensity of human astrocyte binding (PTM-6 and PTM-12; Fig. 5B) and one rhAb that does not bind astrocytes as a negative control (PTM-8). Following this incubation, the percentage of cells with G3BP1^+^ puncta was measured as an indication of astrocyte stress, and the percentage of Caspase 3/7 + cells was measured as an indication of astrocyte toxicity (Fig. 6). We used heatshock as a known inducer of G3BP1 punctate formation to provide a threshold of expected astrocyte stress [45]. Indeed, primary human astrocytes incubated with the negative control rhAb displayed a 2.5-fold increase of G3BP1^+^ puncta following heatshock (p = 0.012) (Fig. 6C). Incubation of human astrocytes with PTM-6 rhAb resulted in a sixfold greater frequency of cells with G3BP1^+^ puncta than the negative control rhAb (p = 0.001) (Fig. 6C). Incubation of human astrocytes with PTM-12 rhAb resulted in a fourfold greater frequency of cells with G3BP1^+^ puncta than the control (p = 0.001) (Fig. 6C). Heatshock treatment did not further increase the frequency of G3BP1 + puncta exposed to PTM-6 or PTM-12 (Fig. 6C). While both PTM-6 and PTM-12 induced astrocytic stress, neither rhAb induced astrocytic toxicity as measured by the frequency of Caspase 3/7 + cells (Fig. 6D).

## Discussion

Patients of any age experiencing initial acute transverse myelitis symptoms are at a critical point when intervention could impact prognosis [23, 37]. For example, use of immunosuppressive therapeutics such as corticosteroids and plasma exchange have shown efficacy in the adult ATM population [48]. However, the use of immunotherapy in the pediatric population could introduce additional issues with extended use. For example, prolonged use of corticosteroids can lead to bone, skin and endocrine complications particularly in pediatric populations [5]. Thus, a deeper understanding of the biology at these early stages of disease in the pediatric ATM population could reveal which pediatric ATM cases would benefit from immunosuppressive therapeutics. We previously demonstrated that adult ATM patients display an expansion of VH4 + PB in the blood, producing antibodies that bind to neurons. The initial focus here was to determine if these same features are displayed in pediatric ATM patients. We began our profiling of pediatric ATM by comparing the frequency of peripheral B cell subsets in pediatric ATM and HC (Fig. 1). In this analysis, we observed that the frequency of PB was significantly reduced in the pediatric ATM cohort compared to pediatric HC (p = 0.0007). One explanation for this observation is that PBs in pediatric ATM cases are not readily detected in the blood because they have extravasated to the central nervous system, presumably in response to inflammation in this compartment. However, we further observed that the total PB population in pediatric ATM was enriched for IgM + PB resulting in a ratio of switched:unswitched PB that was significantly higher in pediatric HC compared to pediatric ATM. This finding was further confirmed using FlowSOM analysis. Thus, the frequency of class-switched PBs in particular is reduced in the blood of pediatric ATM. Others have demonstrated that class-switched (IgA+) PB suppress CNS inflammation in mouse models of MS [40], which may support the notion that suppression of class-switched subsets allows for the CNS inflammation displayed in pediatric ATM patients. However, none of these pediatric ATM cases advanced to MS diagnosis within the 2 years of this study, despite reduced frequency of class-switched PBs. Future investigations should thus focus on the contribution of PB subsets (IgM + versus class-switched) and longitudinal profiling in pediatric ATM which is likely crucial to understanding the potential cause of a first (and perhaps only) ATM episode versus conversion to life-long autoimmune disease (expected 22%) [8, 18].

We next sought to investigate whether this reduced PB population in pediatric ATM maintained its capacity to produce autoreactive immunoglobulin. To address this, we investigated the binding potential of purified IgG from serum samples (Fig. 2) and rhAbs cloned from individual PBs (Figs. 3, 4, 5). We had expected to observe substantial neuronal binding by purified IgG and rhAbs from pediatric ATM patients compared to pediatric HC as an extension of our adult ATM patient data observations. Instead, these two populations displayed similar frequencies and binding intensities to neurons in all tissue types tested. However, in EAE spinal cord tissue, we observed that purified IgG from pediatric HC had an increased mean MFI of binding to astrocytes compared to purified IgG from pediatric ATM. Astrocyte binding was also similar in these two populations in WT tissue samples. To examine this at the single cell level, we cloned 20 antibodies expressed by single PB from the cohort and found that 11 of them bound to astrocytes in EAE spinal cord. The 3 with the highest MFI were from pediatric ATM patients with high accumulation of SHM. A similar observation was made with EAE brain tissue with an enrichment of astrocyte binding by rhAbs that had high accumulation of SHM. While astrocyte reactivity of most of the rhAbs was confirmed in human primary astrocytes (Fig. 5), we did not observe any features of immunogenetics that inferred binding as both VH3 + and VH4 + rhAbs bound human primary astrocytes independent of SHM accumulation. Also, while the 5 rhAbs from the pediatric ATM PBs bound to astrocytes with a similar cytoplasmic pattern, one of the 3 astrocyte binding rhAbs from the pediatric HC cohort (PHC-5) bound exclusively in the nucleus of human primary astrocytes, not the cytoplasm. The PHC-5 rhAb and purified IgG from this same pediatric HC subject displayed typical astrocytic binding in the EAE spinal cord and brain tissue. Of note, the PHC-5 rhAb contains the 3 codon motifs associated with astrocyte binding by 5 of the pediatric TM rhAbs and the other 2 pediatric HC rhAbs, suggesting that other immunogenetic features may contribute to binding of PHC-5 rhAbs to the specific antigen target located in the nucleus. Curiously, none of these three motifs were in codon positions that had undergone SHM but were instead, original to the germline configuration in conserved framework regions of the variable domain. Future investigations should focus on (1) elucidating the impact of these 3 motifs on astrocyte binding properties, and (2) identifying the antigen targets as the binding patterns of these astrocyte-binding rhAbs are distinct.

The PTM-6 and PTM-12 rhAbs displayed the highest MFI of the human primary astrocyte binders (Fig. 5). To address the impact of these rhAbs on astrocyte function, we incubated human primary astrocytes with these two rhAbs and calculated the frequency of human primary astrocytes with G3BP1-marked cytoplasmic puncta as an indication of astrocytic stress [16], and Caspase 3/7 + human primary astrocytes as an indication of apoptotic events (Fig. 6) [45]. Our results indicated that incubation with PTM-6 and PTM-12 alone induced astrocytic stress but did not advance the human primary astrocytes to apoptosis. Subjecting the human primary astrocytes to a heatshock-induced stress event following incubation with PTM-6 did not further exacerbate astrocytic stress and did not advance the human primary astrocytes to apoptosis. Of note, PTM-6 displayed a higher MFI of human astrocyte binding than PTM-12, which may have facilitated its ability to induce a greater astrocytic stress response compared to PTM-12. A comparison of antibodies derived from pediatric ATM subjects to those derived from HC subjects (such as PHC-5 and PHC-6) may clarify whether induction of stress is common to the codon motifs associated with astrocyte binding, or distinct to pediatric ATM subjects.

Stress granules are membrane-less organelles that assemble during stress, injury, and neurological disease [20, 42, 47]. They consist of stalled mRNA transcripts, translation factors, ribosomal subunits, RNA-binding proteins, as well as G3BP1 which is a core component and required for their assembly [53]. While they are thought to be an adaptive response to unfavorable conditions and are normally cleared upon resolution of stress, their chronic presence is cytotoxic in certain cell types [55]. Exposure to IgG against a particular target can induce stress granules in neurons resulting in death [27], but this is the first report demonstrating that exposure to IgG can induce stress granules in astrocytes, but does not lead to death. In future studies, it would be valuable to determine whether chronic exposure to astrocyte-binding antibodies is cytotoxic, or whether presence of complement is required to induce cytotoxicity [32]. Examination of other mechanistic pathways in live-cell incubation assays will also determine the pathogenicity of astrocyte-binding antibodies [21].

The impact of this stress response by astrocytes mediated by astrocyte-binding antibodies on CNS health remains unknown. Astrocytes are essential to BBB integrity [25] and outnumber neurons 50:1 [50]. Astrocytes also transport nutrients to neurons and oligodendrocytes, and astrocyte activation after antibody binding [10] can induce production of cytotoxic factors and enhance immune responses, ultimately forming glial scars that impair remyelination and axonal regeneration [12, 26]. Astrocyte release of inflammatory cytokines disrupts the BBB in MS by activating endothelial cells [1, 34], so astrocyte-binding autoantibodies could challenge BBB integrity and affect entire regions of neurons as observed in NMO with autoantibodies against AQP4 [41, 54]. Given these potentially deleterious effects of astrocyte-binding antibodies on astrocyte function, it stands to reason that it would be beneficial to suppress PB expansion in pediatric ATM patients experiencing CNS inflammation to reduce further injury caused by astrocyte-binding antibodies that PBs from pediatric ATM patients could potentially produce.

However, this conclusion does not resolve the observation that pediatric HC subjects display increased astrocyte binding of purified IgG from serum, and antibodies expressed by individual PB from pediatric HC subjects also bind astrocytes. It is possible that this dichotomy may relate to emerging data suggesting that astrocyte engagement is an important mechanism to reduce neuroinflammation and initiate/sustain neuroprotection [11, 33]**.** This concept was originally established by observations that ischemic preconditioning (IPC) promotes neuroprotection from stroke through mechanisms involving astrocyte activation, including mild responses that would be deleterious to cellular health if larger in magnitude [24]. For example, astrocyte release of nuclear factor erythroid-derived 2-related factor 2 (Nrf2) following preconditioning resulted in neuroprotection from neuroinflammation associated with stroke [17]. Astrocyte activation through the P2X7 receptor has also been strongly implicated in neuroprotection from stroke [19] and astrocyte activation is important in recovery from traumatic brain injury [36]. In the context of Multiple Sclerosis, astrocyte activation through Oncostatin M release activates a feed-forward loop that reduces disease severity in a mouse model of MS [51]. Astrocytes can also modulate both acute and progressive mouse models of MS through a mechanism involving PD-L1 [31].

None of these previous studies investigated the impact of astrocyte-binding antibodies on astrocyte activation. We present evidence here that exposure of astrocytes to astrocyte-binding rhAbs induces astrocytic stress, but not astrocytic death. This raises the intriguing possibility that astrocyte-binding rhAbs may induce a stress response similar to the paradigm of ischemic preconditioning as a pathway to neuroprotection. This study is limited in that (1) we did not have access to cells in the CSF for further study of CNS autoimmunity in pediatric cases, (2) the impact of multiple vaccinations and increased frequency of viral illnesses during childhood is not considered and (3) antigen targets were not identified. Nevertheless, our observations of increased plasmablast frequencies in pediatric HC, increased titers of serum-derived IgG from pediatric HC that bind astrocytes, and increased astrocyte reactivity by rhAbs in the pediatric HC population without the need for SHM to drive astrocyte reactivity all support the concept that astrocyte-binding rhAbs may promote a neuroprotective astrocyte population at early stages of life. Indeed, the differences between pediatric ATM and adult ATM B cell repertoires may suggest that the two immune mediated spinal cord pathologies have distinct pathogenensis and should be studied separately. Such subjects would be protected from clinical manifestations of neuroinflammatory disease, whereas those with a less robust astrocyte-focused humoral immune response (as observed in our pediatric ATM patients using purified IgG pools) would manifest neuroinflammatory disease. To our knowledge, none of the current immunotherapies used in the treatment of pediatric ATM are designed to specifically modulate this potential mechanism. Further investigations of the impact astrocyte-binding antibodies have on astrocyte activation would further elucidate this potential mechanism of neuroprotection and lead to novel therapies specifically beneficial to the pediatric population.

## Conclusions

Pediatric ATM patients display reduced frequency of peripheral PB and reduced titer of astrocyte-binding IgG purified from serum samples compared to pediatric HC. Nevertheless, astrocyte-binding antibodies expressed by PBs identified from pediatric ATM patients induce an astrocyte stress response, but not astrocytic apoptosis. The downstream impact of this astrocytic response has not been elucidated, but introduces an intriguing possibility of antibody-mediated neuroprotection.

### Supplementary Information

Supplementary Material 1.

Supplementary Material 2.

## Data Availability

Data are available upon request to the corresponding author.
