# Supplementary material for: Astrocytic stress response is induced by exposure to astrocyte-binding antibodies expressed by plasmablasts from pediatric patients with acute transverse myelitis
Source: J Neuroinflammation. 2024 Jun 24;21:161. doi: 10.1186/s12974-024-03127-2 (PMC11197286; doi:10.1186/s12974-024-03127-2)

Supplementary Figure S1: CyTOF gating strategy.

Panel A. Cytof manual gates that outline B cell subsets in Panels D-G

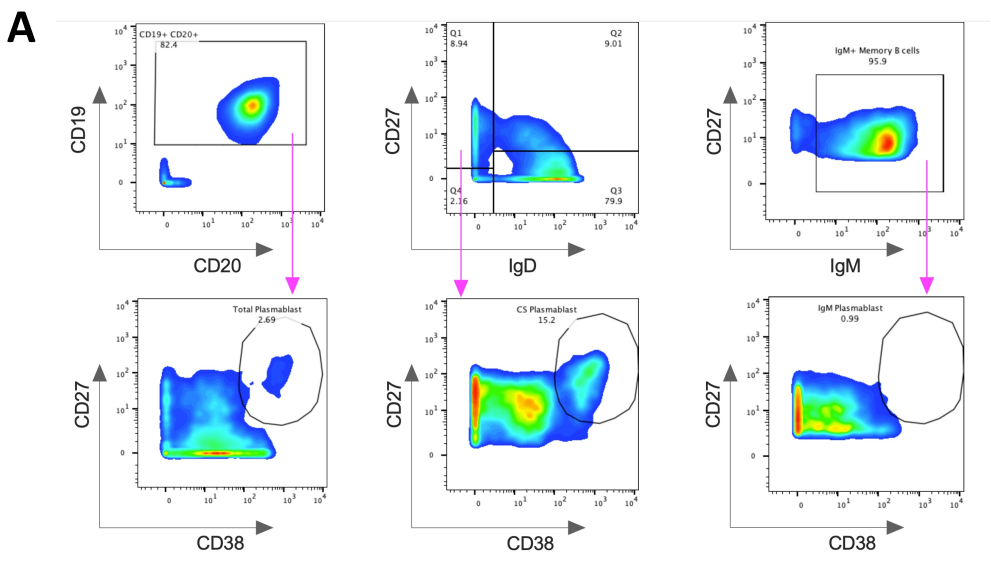

Panel B. CYTOF manual gates that outline B cell subsets in Panels H|I

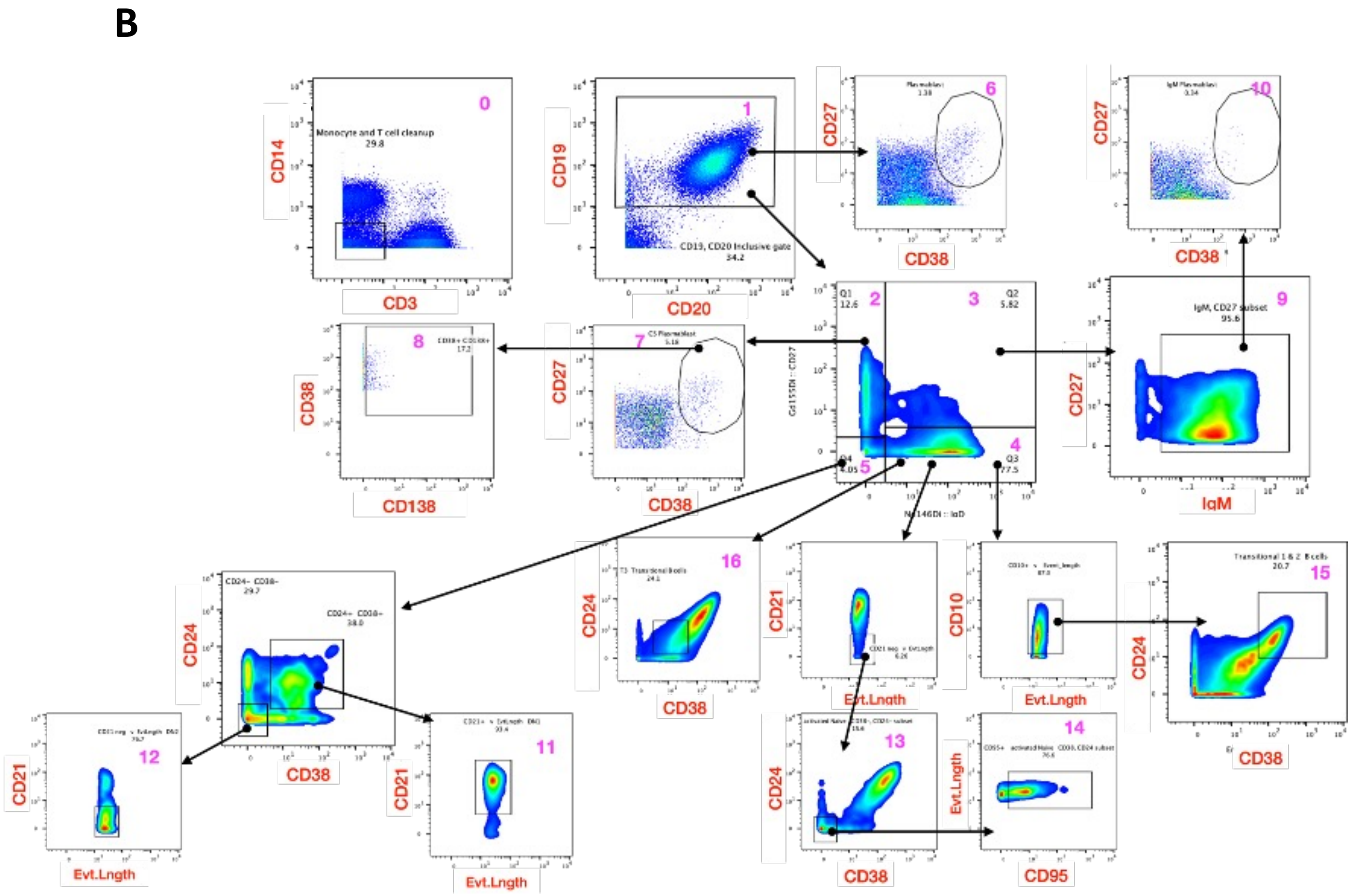

Supplementary Figure S2 – Wild-type and EAE mouse spinal cord reactivity of serum IgG by immunofluorescence.

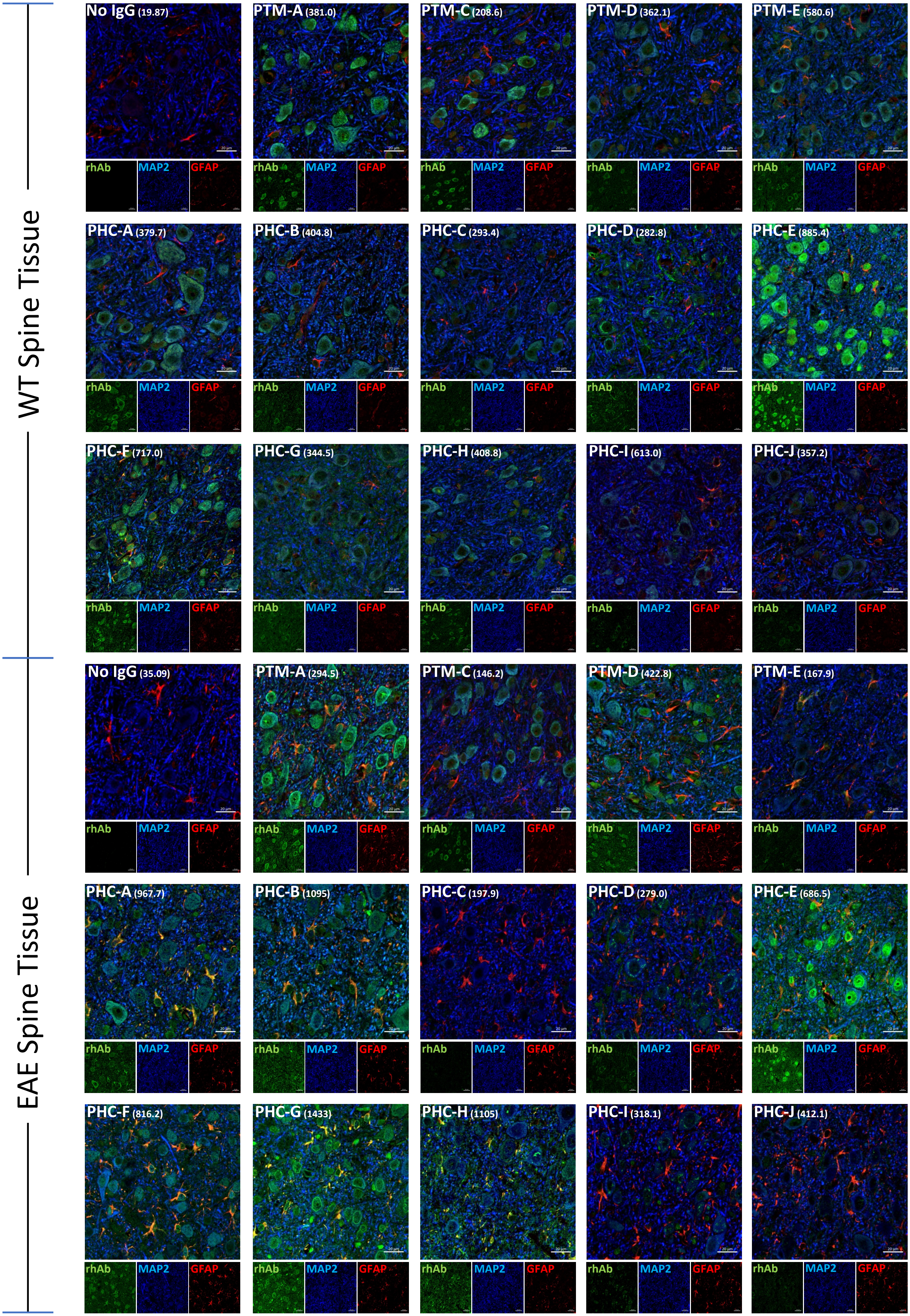

Supplementary Figure S3: Wild-type mouse spinal cord reactivity of rhAbs by immunofluorescence.

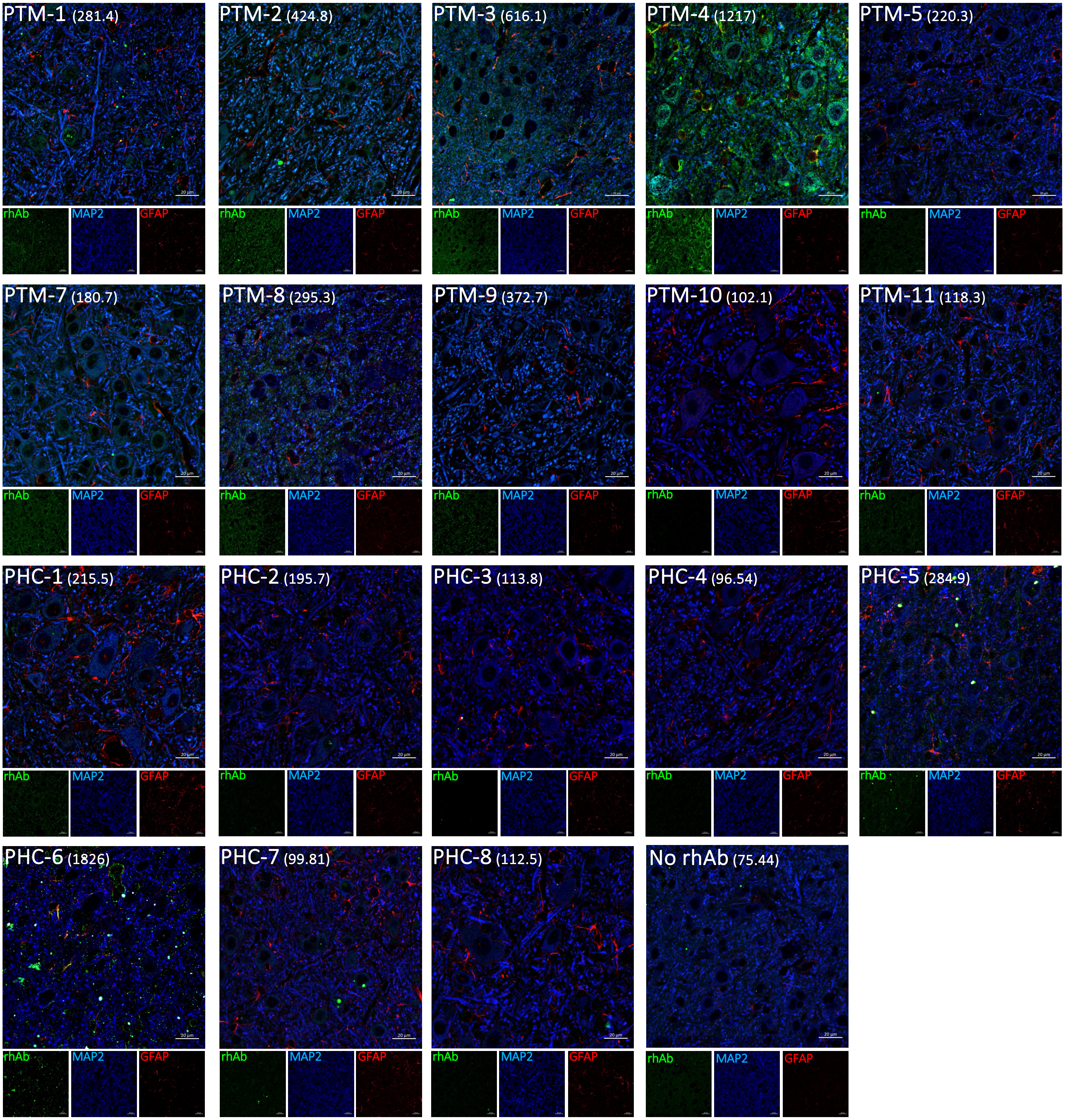

Supplemental Figure S4: EAE mouse spinal cord reactivity of rhAbs by immunofluorescence.

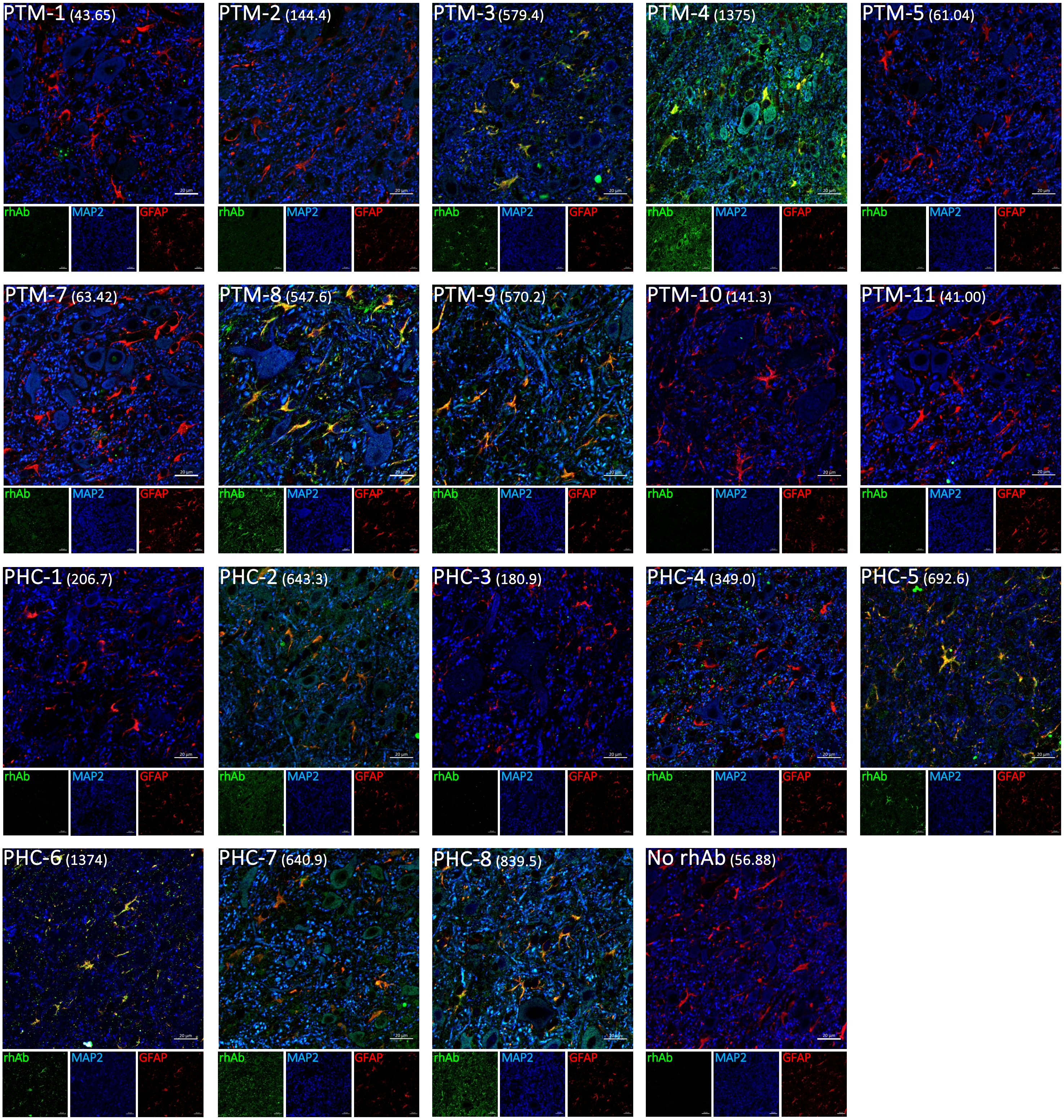

Supplemental Figure S5: Wild-type mouse brain reactivity of rhAbs by immunofluorescence.

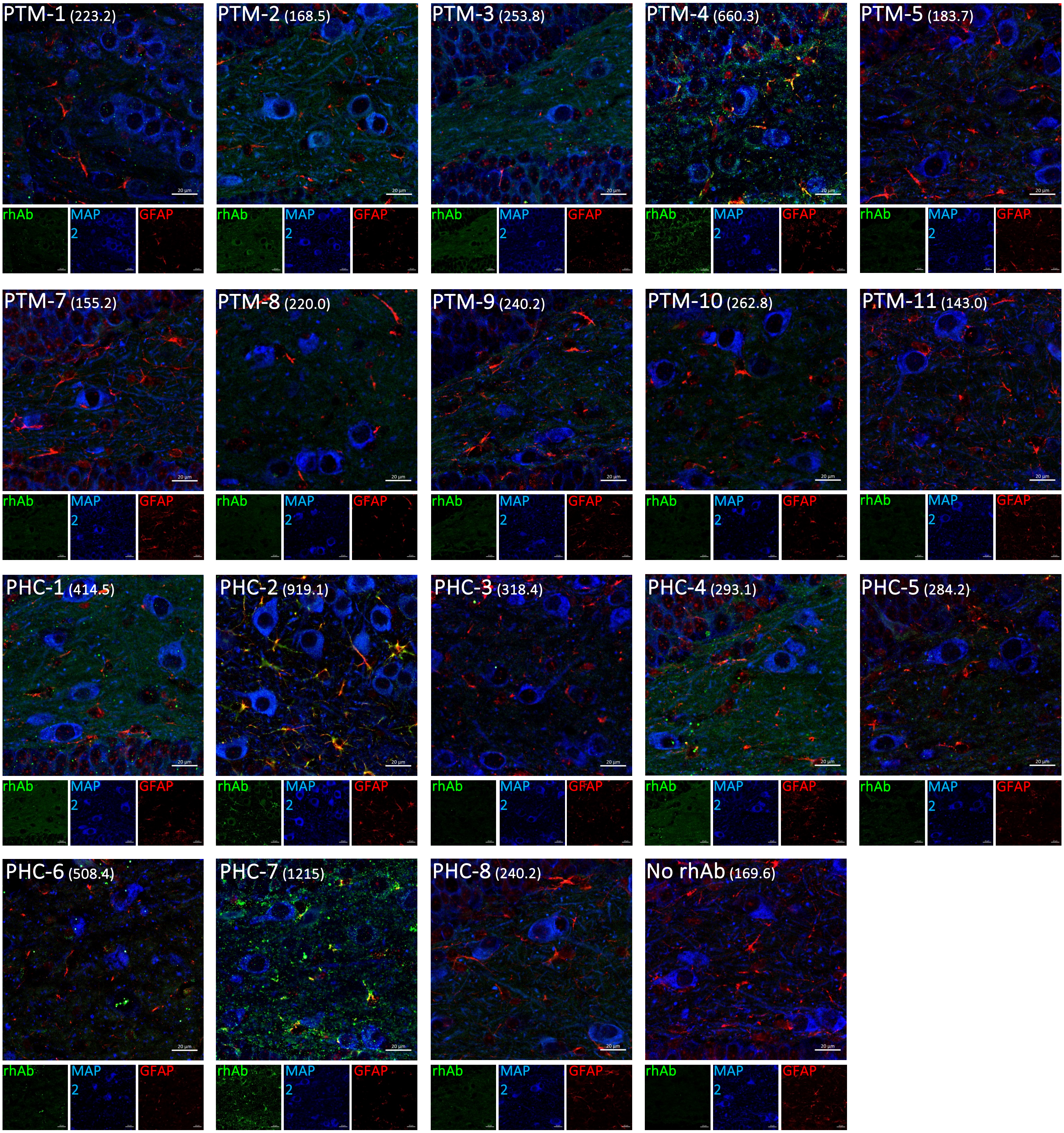

Supplemental Figure S6: EAE mouse brain reactivity of rhAbs by immunofluorescence

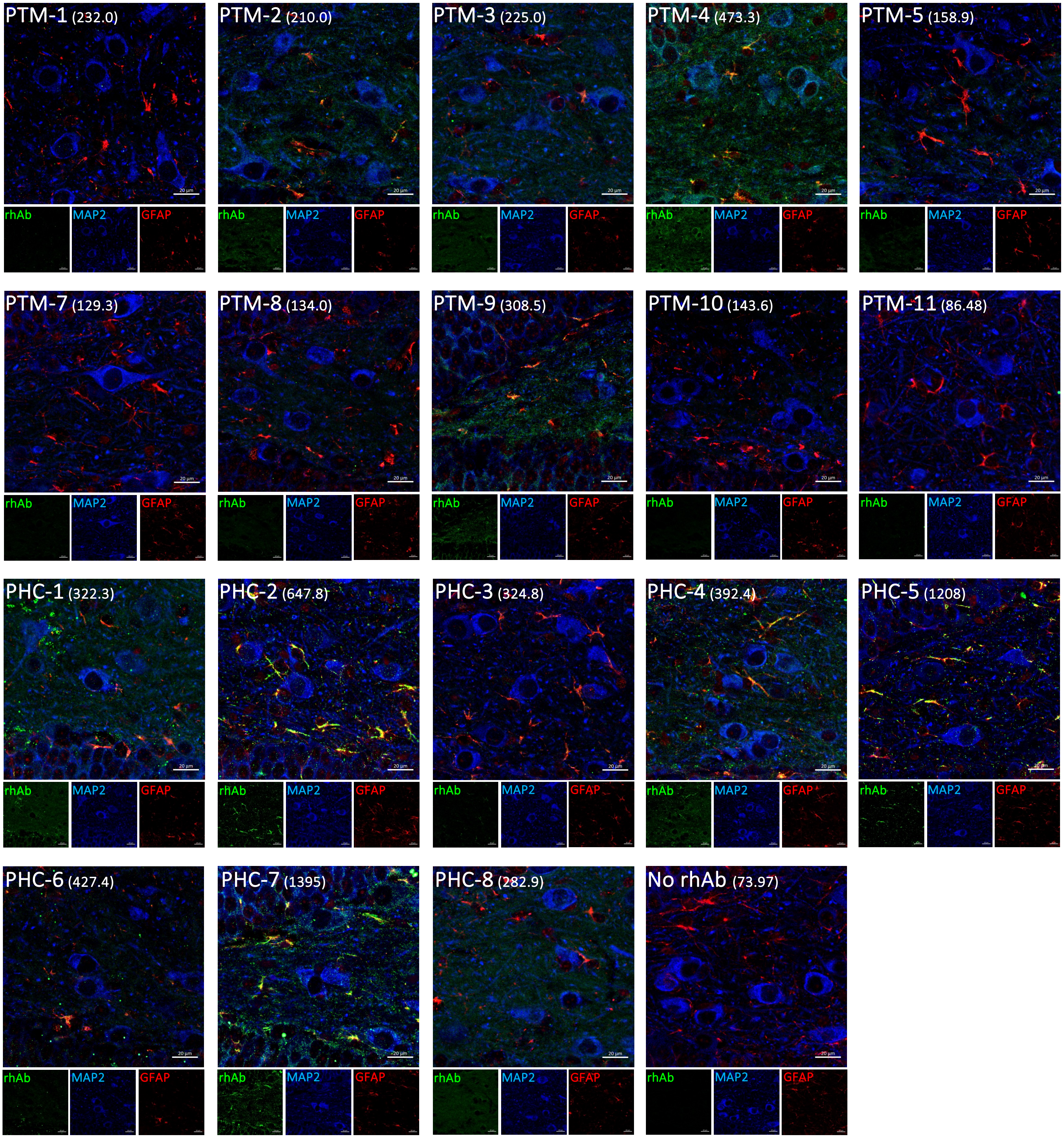

Supplemental Figure S7: Primary human astrocyte reactivity of rhAbs by ICC

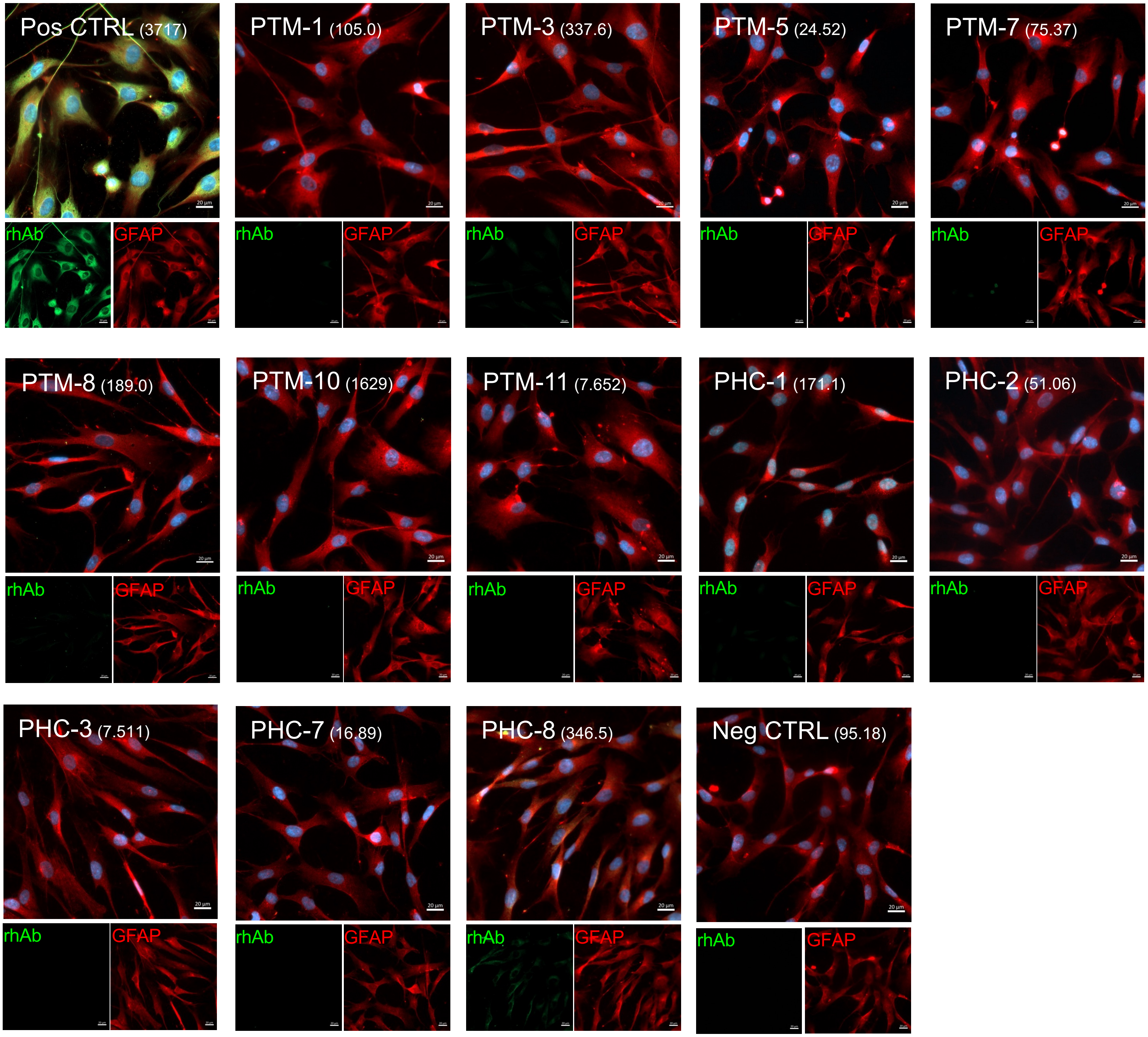

Supplemental Figure S8: Hep2 reactivity of rhAbs by cell-based assay.

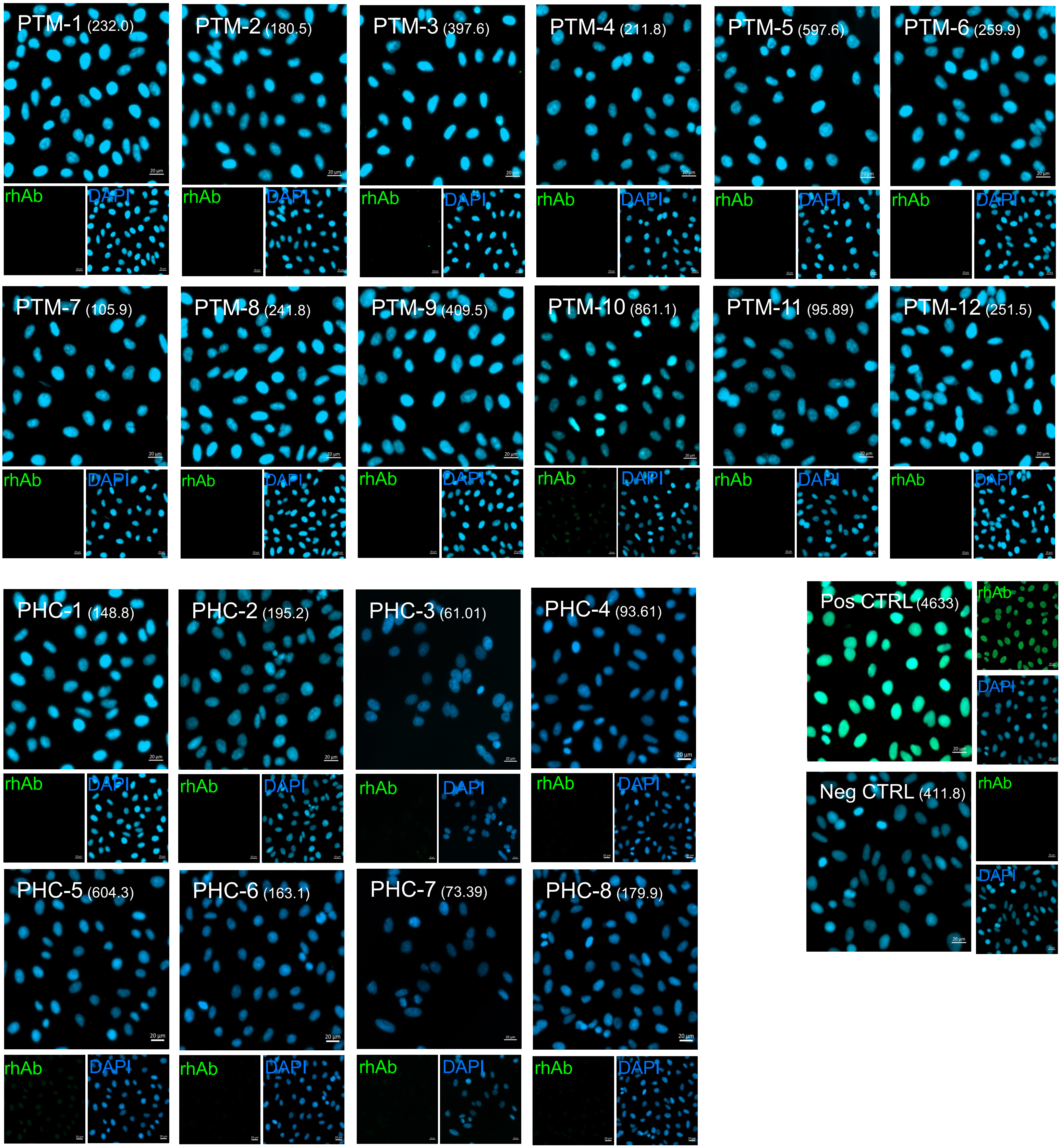

Supplemental Figure S9: Reactivity of rhAbs to HEK293 cells expressing AQP4.

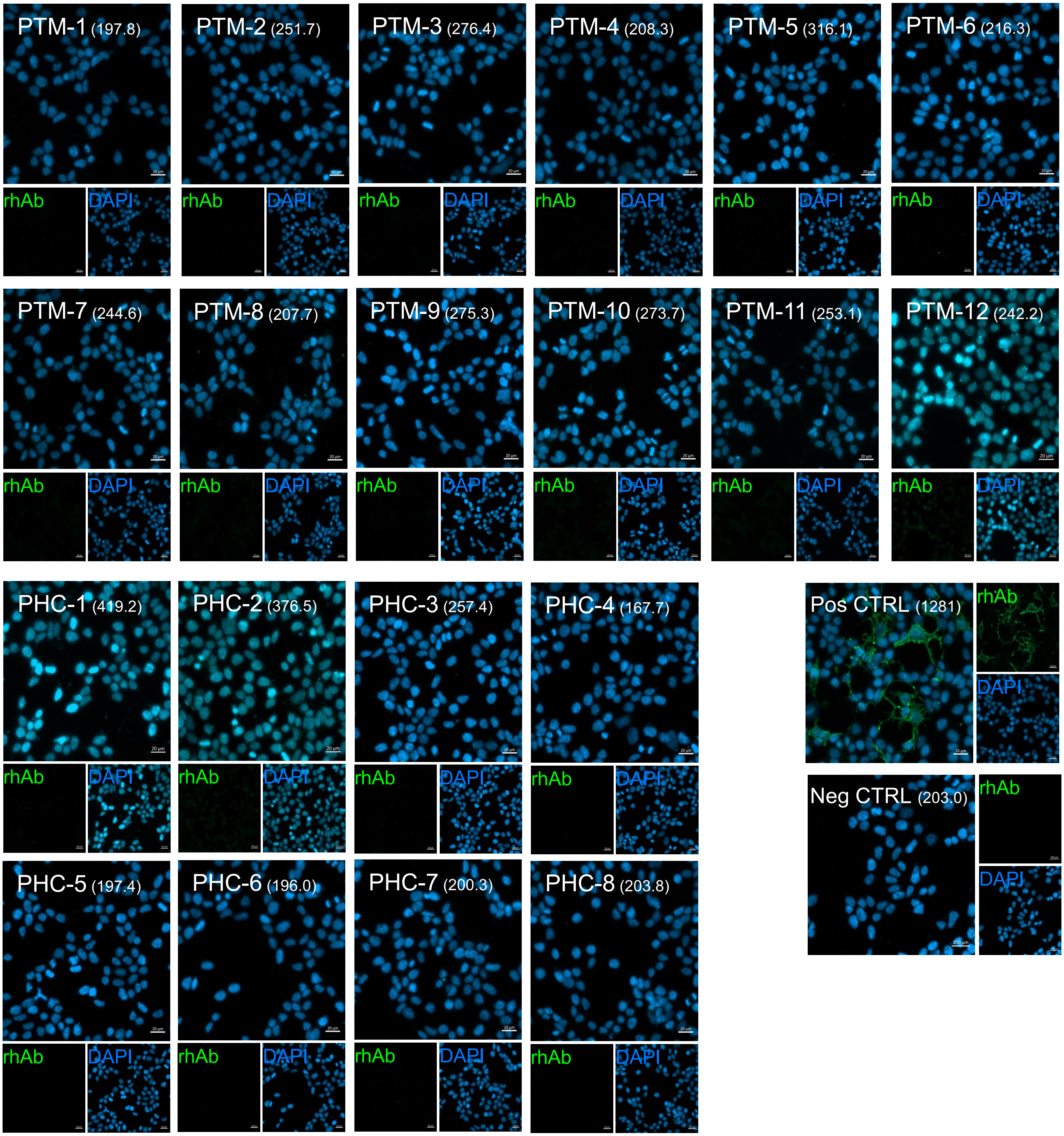

Supplementary Figure S10: rhAbs are internalized to live primary human astrocytes

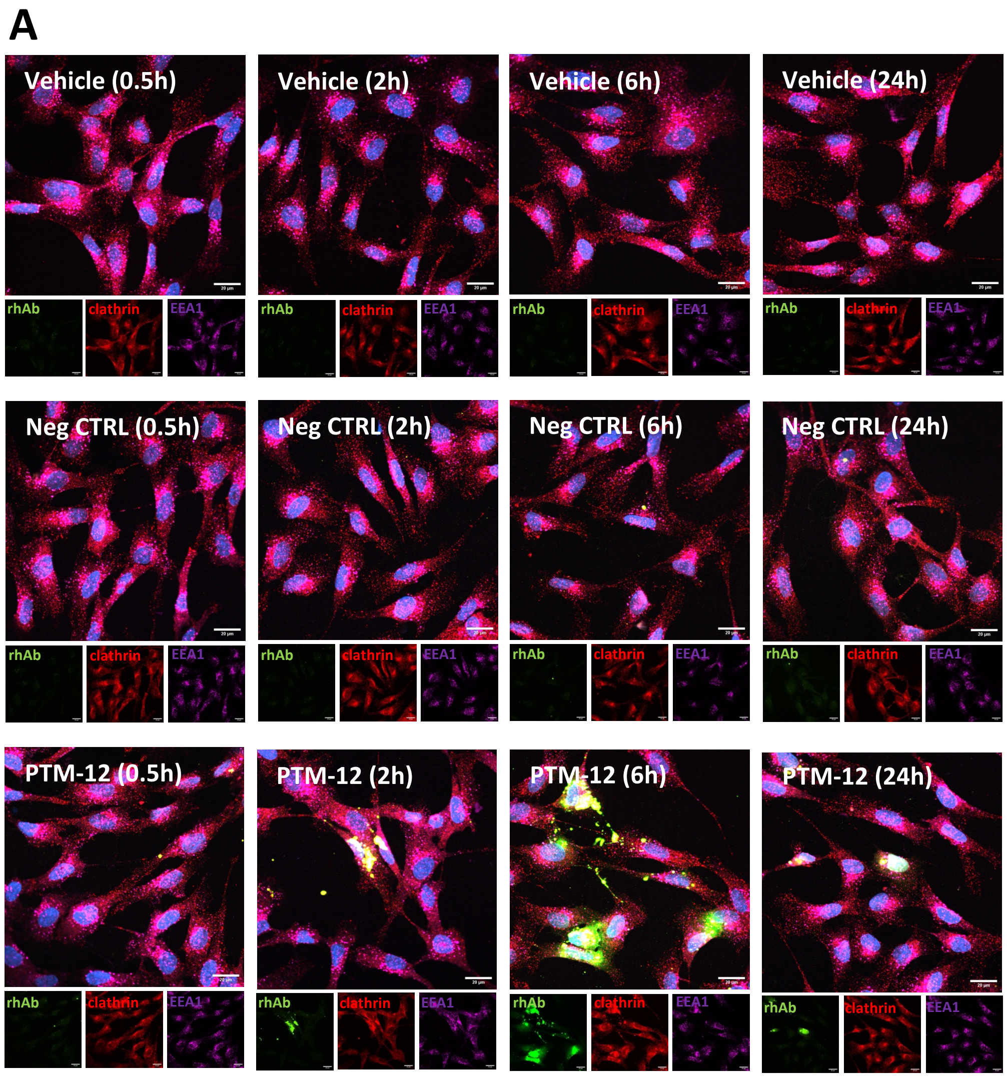

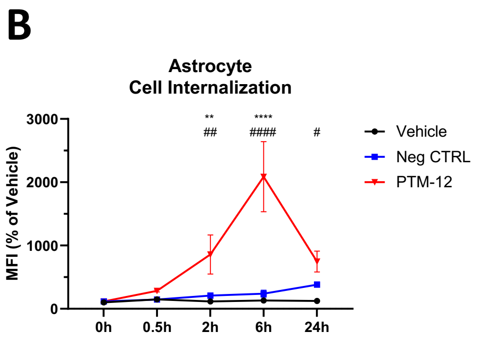

Supplement: Supplementary file 1 — Supplementary Material 1. [file 12974_2024_3127_MOESM1_ESM.pdf]
