# Supplementary material for: Astrocytic stress response is induced by exposure to astrocyte-binding antibodies expressed by plasmablasts from pediatric patients with acute transverse myelitis
Source: J Neuroinflammation. 2024 Jun 24;21:161. doi: 10.1186/s12974-024-03127-2 (PMC11197286; doi:10.1186/s12974-024-03127-2)
Supplement: Supplementary file 2 — Supplementary Material 2. [file 12974_2024_3127_MOESM2_ESM.docx]

| **Supplemental Table 1: B cell subsets identified by CyTOF and marker designations** | | |
| --- | --- | --- |
| **Population #** | **Name** | **Marker Designations** |
| **0** | Monocyte and T cell clean-up |  |
| **1** | **Total** B cells | CD19+ CD20+ (CD3- CD14-) |
| **2** | Q1.   Class switched memory | CD19+ CD20+ CD27+ IgD- |
| **3** | Q2.   Unswitched memory | CD19+ CD20+ CD27+ IgD+ |
| **4** | Q3. **Total** Naive/Transitional B cells | CD19+ CD20+ CD27- IgD+ |
| **5** | Q4.  **Total** Double Negative | CD19+ CD20+ CD27- IgD- |
| **6** | **Total** Plasmablast | CD19+ CD20+ CD27hi CD38+ |
| **7** | Class-switched Plasmablast | CD19+ CD20+ CD27+ IgD- CD27hi CD38+ |
| **8** | CD138+ Class-switched Plasmablast | CD19+ CD20+ CD27+ IgD- CD27hi CD38+ CD138+ |
| **9** | IgM memory | CD19+ CD20+ CD27+ IgD- IgM+ |
| **10** | IgM Plasmablast | CD19+ CD20+ CD27+ IgD- IgM+ CD27hi CD38+ |
| **11** | DN1 | CD19+ CD20+ CD27- IgD- CD24+CD38+ CD21+ |
| **12** | DN2 | CD19+ CD20+ CD27- IgD- CD24- CD38- CD21- |
| **13** | Activated Naive | CD19+ CD20+ CD27- IgD+ CD24- CD38- CD21- |
| **14** | CD95+ Activated Naive | CD19+ CD20+ CD27- IgD+ CD24- CD38- CD21- CD95+ |
| **15** | T1/T2.  Transitional | CD19+ CD20+ CD27- IgD+ CD10+ CD24hi CD38hi |
| **16** | T3.  Transitional | CD19+ CD20+ CD27- IgD+ CD24+ CD38+ |

| **Supplemental Table 2: Summary of rhAb reactivity** | | | | | | | | | | |
| --- | --- | --- | --- | --- | --- | --- | --- | --- | --- | --- |
|  |  |  |  | FIGURE 3 | | FIGURE 4 | | FIGURE 5 | SUPP FIGURE 8 | SUPP FIGURE 9 |
|  |  |  |  | Cord | | Brain | |  |  |  |
| SUBJECT Code | rhAb  Code | Family | Mutations | WT | EAE | WT | EAE | AST | HEP-2 | AQP-4 |
| PHC-B | PHC-1 | VH3 | 0 | N-A- | N-A- | N-A- | N-A+ | NEG | NEG | NEG |
| PHC-D | PHC-2 | VH3 | 0 | N-A- | N+A+ | N+A+ | N+A+ | NEG | NEG | NEG |
| PTM-A | PTM-1 | VH3 | 0 | N-A- | N-A- | N-A- | N-A- | NEG | NEG | NEG |
| PTM-A | PTM-2 | VH3 | 0 | N+A- | N-A- | N-A- | N-A- | POS | NEG | NEG |
| PTM-A | PTM-3 | VH3 | 0 | N-A- | N-A+ | N-A- | N-A- | NEG | NEG | NEG |
|  |  |  |  |  |  |  |  |  |  |  |
| PHC-A | PHC-3 | VH3 | 14 | N-A- | N-A- | N-A- | N-A+ | NEG | NEG | NEG |
| PHC-E | PHC-4 | VH3 | 11 | N-A- | N-A- | N-A- | N+A+ | POS | NEG | NEG |
| PTM-A | PTM-4 | VH3 | 10 | N+A+ | N+A+ | N+A+ | N+A+ | POS | NEG | NEG |
| PTM-A | PTM-5 | VH3 | 6 | N-A- | N-A- | N-A- | N-A- | NEG | NEG | NEG |
| PTM-B | PTM-6 | VH3 | 9 | N-A+ | N-A+ | N+A+ | N+A+ | POS | NEG | NEG |
|  |  |  |  |  |  |  |  |  |  |  |
| PHC-B | PHC-5 | VH4 | 0 | N-A- | N-A+ | N-A- | N-A+ | POS | NEG | NEG |
| PHC-D | PHC-6 | VH4 | 0 | N-A+ | N-A+ | N-A- | N-A+ | POS | NEG | NEG |
| PTM-A | PTM-7 | VH4 | 0 | N-A- | N-A- | N-A- | N-A- | NEG | NEG | NEG |
| PTM-A | PTM-8 | VH4 | 0 | N-A- | N-A+ | N-A- | N-A- | NEG | NEG | NEG |
| PTM-B | PTM-9 | VH4 | 0 | N-A- | N+A+ | N-A- | N-A+ | POS | NEG | NEG |
|  |  |  |  |  |  |  |  |  |  |  |
| PHC-E | PHC-7 | VH4 | 11 | N-A- | N+A+ | N+A+ | N+A+ | NEG | NEG | NEG |
| PHC-A | PHC-8 | VH4 | 5 | N-A- | N+A+ | N-A- | N+A+ | NEG | NEG | NEG |
| PTM-A | PTM-10 | VH4 | 15 | N-A- | N-A- | N-A- | N-A- | NEG | NEG | NEG |
| PTM-A | PTM-11 | VH4 | 15 | N-A- | N-A- | N-A- | N-A- | NEG | NEG | NEG |
| PTM-C | PTM-12 | VH4 | 5 | N-A- | N-A+ | N-A+ | N-A+ | POS | NEG | NEG |

**Supplementary Figure S1: CyTOF gating strategy. (A)** CyTOF manual gating outlining B cell subsets in Figure 1D-G. **(B)** CyTOF manual gating outlining B cell subsets in Figure 1H-I.

**Supplementary Figure S2: Wild-type and EAE mouse spinal cord reactivity of serum IgG by immunofluorescence.** Representative images from the lumbar spinal cord of WT (top) and EAE (bottom) mice. Green: IgG staining. Red: GFAP. Blue: MAP2. In the merge panels, yellow indicates co-stain of GFAP with the rhAb. The MFI of astrocytic rhAb signal is indicated in parentheses. Scale bar: 20μm.

**Supplementary Figure S3: Wild-type mouse spinal cord reactivity of rhAbs by immunofluorescence.** Representative images from the lumbar spinal cord of WT mice. Green: IgG staining. Red: GFAP. Blue: MAP2. In the merge panels, yellow indicates co-stain of GFAP with the rhAb and. The MFI of astrocytic rhAb signal is indicated in parentheses. Scale bar: 20μm.

**Supplemental Figure S4: EAE mouse spinal cord reactivity of rhAbs by immunofluorescence.** Representative images from the lumbar spinal cord of EAE mice. Green: IgG staining. Red: GFAP. Blue: MAP2. In the merge panels, yellow indicates co-stain of GFAP with the rhAb. The MFI of astrocytic rhAb signal is indicated in parentheses. Scale bar: 20μm.

**Supplemental Figure S5: Wild-type mouse brain reactivity of rhAbs by immunofluorescence.** Representative images from the hippocampus of WT mice. Green: rhAb staining. Red: GFAP. Blue: MAP2. In the merge panels, yellow indicates co-stain of GFAP with the. The MFI of astrocytic signal is indicated in parentheses. Scale bar: 20μm.

**Supplemental Figure S6: EAE mouse brain reactivity of rhAbs by immunofluorescence.** Representative images from the hippocampus of EAE mice. Green: rhAb staining. Red: GFAP. Blue: MAP2. In the merge panels, yellow indicates co-stain of GFAP with the rhAb. The MFI of astrocytic rhAb signal is indicated in parentheses. Scale bar: 20μm.

**Supplemental Figure S7: Primary human astrocyte reactivity of rhAbs by ICC.** Representative images from primary human astrocyte cultures. Green: rhAb staining. Red: GFAP. Blue: DAPI. In the merge panels, yellow indicates co-stain of GFAP with the rhAb. The MFI of astrocytic rhAb signal is indicated in parentheses. Scale bar: 20μm.

**Supplemental Figure S8: Hep2 reactivity of rhAbs by cell-based assay.** Representative images from Hep2 cell-based assay. Green: rhAb and positive control staining. Blue: DAPI. The MFI of Hep2 rhAb signal is indicated in parentheses. Scale bar: 20μm.

**Supplemental Figure S9: Reactivity of rhAbs to HEK293 cells expressing AQP4.** Representative images from anti-AQP4 cell-based assay. Green: rhAb and positive control staining. Blue: DAPI. The MFI of AQP4-expressing cells is indicated in parentheses. Scale bar: 20 μm.

**Supplementary Figure S10: rhAbs are internalized to live primary human astrocytes. (A)** Representative images of human astrocyte cell cultures incubated with rhAbs for 30m, 2h, 6h and 24h. Green: rhAb staining. Red: Clathrin heavy chain. Violet: EEA1. Blue: DAPI. Scale bar: 20 μm. **(B)** Graphs quantifying rhAb fluorescence intensity in the entire cell. * p < 0.05, ** p < 0.01, *** p < 0.001, **** p < 0.0005 by Two-Way ANOVA followed by Tukey’s post-test.

Supplemental Method for CyTOF analysis

Semi-Automated Gating Analysis of CyTOF Data Using DAFi

DAFi [PMCID: PMC6030426] is a recursive data filtering and clustering approach for improving and interpreting data clustering identification of cell populations from high-dimensional cytometry data. DAFi requires an input of a manual gating strategy to indicate the location of the cells of interest in 2D dot plots. Data clustering analysis is then applied in a recursive way to identify the cell populations in the high-dimensional space. By integrating the manual gating strategy to constrain and guide the unsupervised clustering process, the results of DAFi are not only data-driven but also straightforward to interpret by immunologists. Boundaries of the DAFi-identified cell populations are not limited by the abrupt cutoffs used in the manual gating strategy, while each of the identified cell populations is visualized in sequential 2D dot plots following the original manual gating strategy for interpretation.

FCSTrans [PMCID: PMC3932304] was applied to transform the CyTOF data before DAFi was applied to analyze all 15 CyTOF FCS files. Both FCSTrans and DAFi are open sourced and can be downloaded at: <https://github.com/JCVenterInstitute/DAFi-gating/>. The gating strategy used in the DAFi analysis identified 26 cell populations. The first 6 DAFi gating steps were for filtering out debris, dead cells, doublets, non-leukocytes, monocytes, and T lymphocytes, based on EQ1, Event_Length, DNA1, DNA2, Cisplatin, CD45, CD3, and CD14. The number of events, percentage of the cell population, as well as the mean fluorescence intensity (MFI) for each cell population identified by DAFi were output for downstream statistical analysis for comparisons between the healthy and the PTM cohort.

Automated Gating Analysis of CyTOF Data Using FlowSOM and Nonlinear Transformation and Visualization of CyTOF Data Using t-SNE

Preprocessing of the FCS files including transformation and manual gating analysis was done on the OMIQ software platform (<https://www.omiq.ai/>). The FCS files were first rescaled by arcsinh transformation (Cofactor=5, (min, max)=(-5, 12000)), followed by a manual gating process on EQ1, Event_Length, DNA1, DNA2, Cisplatin, CD45, CD3, and CD14 to filter out debris, dead cells, doublets, non-leukocytes, monocytes, and T lymphocytes. To both reduce the runtime and normalize the input size, a down-sampling step based on random sampling was applied to equalize the number of events across the samples for meta-clustering analysis and visualization, which reduced the total number of events from 1,342,301 to 314,905, referred to be CD3^-^CD14^-^ live singlets as the input to both t-SNE and FlowSOM analytics.

To visualize the high-dimensional mass cytometry data, nonlinear embedding transformation approaches such as t-SNE (t-distributed stochastic neighbor embedding; van der Maaten L, Hinton G (2008): Visualizing Data using t-SNE. Journal of Machine Learning Research 9 (11): 2579-2605) provide an effective way to extract the data distribution patterns in the high-dimensional space and project them into a 2D map. We used the OMIQ platform to pool the CD3^-^CD14^-^ live singlets of all the 15 samples and input them to a t-SNE run (1000 iterations, learning rate = 5000, perplexity = 30, theta = 0.5), before visualizing each cohort or each sample in individual t-SNE maps.

FlowSOM (analysis of flow or mass cytometry data using a Self-Organizing Map [PMID: 25573116; PMID: 34172973]) is one of the most popular automated gating analysis methods for CyTOF mass cytometry data analysis. We performed the FlowSOM analysis on the OMIQ platform, using the CD3^-^CD14^-^ live singlets as the input to the FlowSOM clustering. A 10-by-10 grid (i.e., 100 initial clusters) was used to initialize the FlowSOM run based on the Euclidean distance, while K=20 was specified for generating 20 meta clusters across the samples using the consensus meta-clustering option in FlowSOM. The 20 meta clusters of cells were then color-coded and visualized on both the t-SNE map and the bivariate dot plots used in the manual gating strategy for cross-sample comparison and interpretation.
